# Long-term impact of fecal transplantation in healthy volunteers

**DOI:** 10.1186/s12866-019-1689-y

**Published:** 2019-12-30

**Authors:** Oleg V. Goloshchapov, Evgenii I. Olekhnovich, Sergey V. Sidorenko, Ivan S. Moiseev, Maxim A. Kucher, Dmitry E. Fedorov, Alexander V. Pavlenko, Alexander I. Manolov, Vladimir V. Gostev, Vladimir A. Veselovsky, Ksenia M. Klimina, Elena S. Kostryukova, Evgeny A. Bakin, Alexander N. Shvetcov, Elvira D. Gumbatova, Ruslana V. Klementeva, Alexander A. Shcherbakov, Margarita V. Gorchakova, Juan José Egozcue, Vera Pawlowsky-Glahn, Maria A. Suvorova, Alexey B. Chukhlovin, Vadim M. Govorun, Elena N. Ilina, Boris V. Afanasyev

**Affiliations:** 1 R.M.Gorbacheva Memorial Institute of Oncology, Hematology and Transplantation, Pavlov First Saint Petersburg State Medical University, St. Petersburg, Russian Federation; 2 Federal Research and Clinical Centre of Physical and Chemical Medicine of Federal Medical and Biological Agency of Russia, Moscow, Russian Federation; 3 Pediatric Research and Clinical Center for Infectious Diseases, St. Petersburg, Russia; 4 Mechnikov North-West State Medical University, St. Petersburg, Russia; 5 Universitat Politècnica de Catalunya, Barcelona, Spain; 6 Universitat de Girona, Girona, Spain; 7 Explana Research Laboratory, St. Petersburg, Russian Federation

**Keywords:** Fecal microbiota transplantation, Healthy volunteers, Metagenomics, 16S rRNA gene sequencing, Shotgun sequencing, Metagenome-assembled genome, Compositional data analysis

## Abstract

**Background:**

Fecal microbiota transplantation (FMT) has been recently approved by FDA for the treatment of refractory recurrent clostridial colitis (rCDI). Success of FTM in treatment of rCDI led to a number of studies investigating the effectiveness of its application in the other gastrointestinal diseases. However, in the majority of studies the effects of FMT were evaluated on the patients with initially altered microbiota. The aim of our study was to estimate effects of FMT on the gut microbiota composition in healthy volunteers and to monitor its long-term outcomes.

**Results:**

We have performed a combined analysis of three healthy volunteers before and after capsule FMT by evaluating their general condition, adverse clinical effects, changes of basic laboratory parameters, and several immune markers. Intestinal microbiota samples were evaluated by 16S rRNA gene and shotgun sequencing. The data analysis demonstrated profound shift towards the donor microbiota taxonomic composition in all volunteers. Following FMT, all the volunteers exhibited gut colonization with donor gut bacteria and persistence of this effect for almost ∼1 year of observation. Transient changes of immune parameters were consistent with suppression of T-cell cytotoxicity. FMT was well tolerated with mild gastrointestinal adverse events, however, one volunteer developed a systemic inflammatory response syndrome.

**Conclusions:**

The FMT leads to significant long-term changes of the gut microbiota in healthy volunteers with the shift towards donor microbiota composition and represents a relatively safe procedure to the recipients without long-term adverse events.

## Background

Human gut microbiota is a key player in human body metabolism. Gut microbiota begins to develop from birth and its composition depends on multiple factors: delivery type, nosocomial microflora at the obstetrics unit, maternal diet, breastfeeding etc. [1, 2]. The microbiota is extremely important for the maintenance of physiological homeostasis including synthesis of vitamins and essential amino acids, short-chain fatty acids (SCFA), e.g., butyrate, propionate, acetate which serve as energy substrates for epithelial cells as well as inactivation of toxic substances [3]. Antibacterial and/or cytostatic treatments trigger profound changes in gut microbiota composition reducing bacterial diversity and increasing predominance of pathogenic microorganisms that facilitate damage to a gut epithelium barrier and/or alter immune system response [4].

Fecal microbiota transplantation (FMT) from allogeneic donors has become a popular approach to the microbiota correction. Recently, the FMT procedure was approved by the FDA (Food and Drug Administration) for application in the setting of clinical trials in recurrent clostridial colitis (recurrent *Clostridium difficile* infection – rCDI) [5]. However, several procedure limitations still exist, thus precluding wider implementation of this technology, especially in other clinical settings [6].

Growing interest to this method is determined by a high response rate (>90%) in rCDI, including cases with multiple antibiotic resistance [7], positive therapeutic effect in severe cases of ulcerative colitis [8], Crohn’s disease [9], as well as by a relatively simple application method. There is also evidence of FMT efficacy in correcting microbiota following antibacterial treatment [10]. A number of published data provide evidence for the effectiveness of FMT in complex therapy of autoimmune diseases [11], antibiotic-associated diarrhea, and in graft-versus-host disease occurring after hematopoietic stem cell transplantation [12, 13]. The FMT procedure results in reduced prevalence of gut *Enterobacteriaceae* with multiple resistance to beta-lactam antibiotics and carbapenems, vancomycin-resistant *Enterococcus* spp. (VRE), methicillin-resistant *Staphylococcus aureus* (MRSA) [14], *Klebsiella pneumoniae* and other drug-resistant bacteria [15–17]. These observations are particularly valuable in the light of the high mortality caused by antibiotic-resistant pathogens [18].

It has been previously shown that FMT induces multiple changes in the gut microbiota composition [19, 20]. The main mechanism of FMT effects in inflammatory bowel diseases (IBD) is believed to be associated with colonization of the gut with donor microbiota [21]. However, there is a lack of data on the exact mechanisms behind FMT efficacy. Kump et al., have shown that the changes in taxonomic spectrum of a donor microbiota is the main factor determining FMT efficacy in patients with ulcerative colitis [22]. On the other hand, colonization with donor microbiota may, of course, promote the metabolic potential of the recipient microflora, thus causing clinical improvement. However, these studies were carried out by treating already severely ill patients, or in vivo animal models. Despite encouraging results with FMT in different clinical settings, we have not found any data on typical effects of FMT in healthy subjects, evaluating and/or tracing the microbiota shifts, and investigating the appropriate immune system responses. These data would allow better understanding of the changes after the treatment in different clinical disorders and may specify changing patterns of the host-donor microbiota interrelations after FMT.

Despite the convincing success of FMT, a number of adverse effects ranging from abdominal complaints to fever, bacteremia, and exacerbation of underlying diseases were reported [23]. However, the data addressing possible FMT complications in healthy volunteers treated by allogeneic microbiota are still lacking. A knowledge of FMT effects in healthy and diseased persons will enable better awareness of participants at future FMT clinical trials seeking for safety and health risk minimization among the study subjects. Hence, the aim of our study was to evaluate effects of FMT upon gut microbiota in healthy persons following FMT from a healthy donor, as well as basic parameters of the immune system before and after this procedure.

## Methods

### Donor and volunteers selection

A woman, in her mid-thirties, was chosen as a donor (body mass = 54 kg, BMI = 19.4). She received a usual, balanced, European diet over the entire period of the study and was clinically evaluated according to a protocol recommended by the European Consensus Conference on Faecal Microbiota Transplantation in Clinical Practice [24]. Firstly, the donor has been good health, being subjected to an annual routine medical checkup. Her physical condition, blood counts and routine serum biochemistry were found to be within normal limits for the preceding 3 years. No dyspepsia or other stool abnormalities were reported within last year. Routine clinical and laboratory examinations such as clinical blood cell counts, biochemical blood analysis, and evaluation of lymphocyte subpopulations showed no abnormalities, as well as human viruses blood testing (such as HIV, hepatitis A-E, CMV, Epstein–Barr virus, HSV-1, HSV-2, HHV-6) were negative. Syphilis blood testing was also negative. Secondly, donor stool has been thoroughly examined: (1) PCR for intestinal microorganisms including opportunistic species (Colonoflor-16, LLC Alfa Lab, Russia); (2) bacteriology for antibiotic resistance such as MRSA, VRE, ESBL, carbapenemase-producing *Klebsiella pneumoniae* and *Escherichia coli*; (3) study of fecal calprotectin; (4) examination for *Clostridium difficile* toxins A and B; (5) PCR testing for enteropathogenic viruses: CMV, Epstein-Barr virus, HSV-1, HSV-2, HHV-6, human adenoviruses (types B, C, E), noroviruses (types 1, 2), rotaviruses (types A, B, C), enterovirus; (6) parasites testing including protozoa and helminths. After 90 days, the repeated blood tests were performed as described above. Implemented clinical testing didn’t show abnormalities. Three consequent donor samples were collected: baseline specimens (used for FMT), as well as 193 and 385 days later.

Full awareness of the volunteers of the study purpose, perform and possible adverse effects from treatment, were strictly followed through, as stated by approved informed consent signed by each participant in this study. The volunteers’ medical information was anonymized. Access to full information was available only to the doctor. Volunteers were physically and mentally healthy. They kept a standard European diet. After the procedure participants were monitored in the outpatient department of R.M. Gorbacheva memorial institute and had access to all services such as bone marrow transplant recipients, including daily care, 24/7 on-call haematologist and opportunity for hospitalization. The summary data on the volunteers and the donor are presented in Additional file 6: Table S1.

### Treatment schedule

**Fig. 1:**
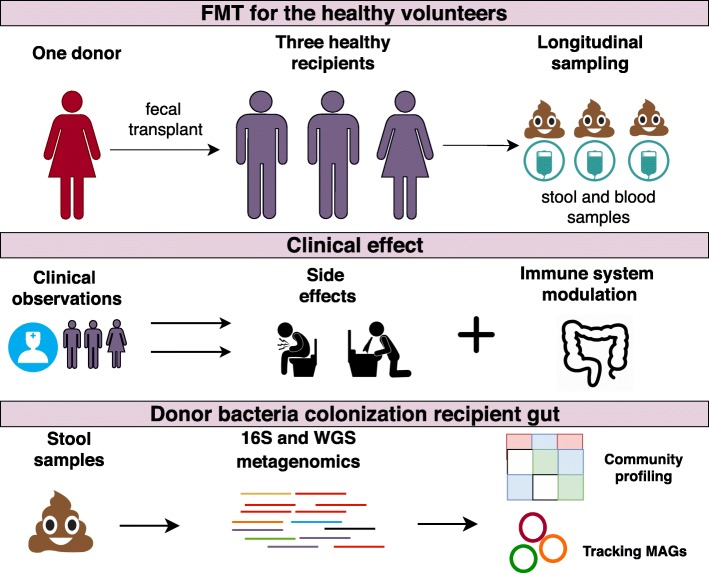
Study description. The first line describes sampling points, the second line clinical effect observable caused by taking FMT capsule. The third line describes obtaining sequencing data and bioinformatic analysis. The sampling and sequencing points are presented in Additional file 6: Table S1

We have used the FMT protocol including dosage and timing of the procedure, as well as observation terms which, in general, correspond to the protocol from the clinical trial NCT03214289. The workflow of the study was used in other clinical trials applied for the treatment of different gastrointestinal disorders [25]. The FMT procedure was performed in three healthy volunteers (38.6 ±7.4 years old, two male and one female). Volunteers were assigned IDs – V1, V2 and V3. The treatment was two-staged: the first stage consisted of the administration of 15 capsules containing donor stool on one day, and 15 capsules on the next day. Mild breakfast was allowed 4 hours before administration. One hour before treatment, each volunteer took a dose of omeprazole (20 mg). The volunteers were administered solid gelatin capsules (Coni-Snap$^{\circledR }$ size 4) containing frozen at -20 ^∘^C feces, followed by drinking water. The anticipated weight of the material for every single volunteer was 22 g in 30 capsules. The clinical follow-up continued for 300-303 days. In total, 22 fecal and blood samples of volunteers were collected. The gut metagenomic study was performed for the first volunteer in ten time points; he underwent two FMTs (the second FMT was carried out 38 days after the first FMT). The second volunteer (V2) was administered half of the anticipated dose due to the development of systemic inflammatory response syndrome (SIRS). The third volunteer was administered the full anticipated dose. Adverse effects (AE) were assessed using the Toxicity Scale (Common Terminology Criteria for Adverse Events (CTCAE) Version 5.0 Published: November 27, 2017). The same protocol of fecal microbiota transplantation was approval and used for patients with refractory *Clostridium difficile* infection and graft-versus-host disease in Pavlov First Saint Petersburg State Medical University hospital practice [26]. The study description scheme presented in Fig. 1.

### Sample collection, preparation and sequencing

Collection of stool samples was performed in sterile plastic containers, both before FMT and at different time points later on. DNA was extracted using PureLink ^*TM*^ Microbiome DNA Purification Kit (Invitrogen ^*TM*^, USA) according to the manufacturer’s protocol. 16S library preparation and sequencing were done according to Illumina protocol (16S Metagenomic Sequencing Library Preparation). Briefly, extracted DNA was amplified using standard 16S rRNA gene primers, complementary to V3-V4 region and containing 5’-illumina adapter sequences. In the next step individual amplicons were PCR – indexed and pooled. DNA libraries were sequenced on a MiSeq instrument (Illumina, San Diego, CA, USA) using Miseq reagent kit v3 (Illumina, San Diego, CA, USA). For shotgun sequencing, 300 ng of DNA were sheared by sonication with the Covaris S220 System (Covaris, Woburn, Massachusetts, USA). The final sizes of fragmented DNA samples were determined on Agilent 2100 Bioanalyzer (Agilent, USA) using the manufacturer guide, and were approximately 400-500 bp long. Paired-end libraries were prepared according to the manufacturer’s recommendations using NEBNext Ultra II DNA Library Prep Kit (New England Biolabs, USA). The libraries were indexed with NEBNext Multiplex Oligos kit for Illumina (96 Index Primers, New England Biolabs, USA). Size distribution for the libraries and their quality were assessed using a high-sensitivity DNA chip (Agilent Technologies). The libraries were subsequently quantified by Quant-iT DNA Assay Kit, High Sensitivity (Thermo Scientific, USA). DNA sequencing was performed on the HiSeq 2500 platform (Illumina, USA) according to the manufacturer’s recommendations, using the following reagent kits: HiSeq Rapid PE Cluster Kit v2, HiSeq Rapid SBS Kit v2 (500 cycles), HiSeq Rapid PE FlowCell v2 and a 2% PhiX spike-in control.

Dynamic monitoring of clinical blood cell counts, biochemical blood analysis, and evaluation of lymphocyte subpopulations was performed in the recipients. Immunophenotyping was performed with a flow cytometer Cytomics FC500 (Beckman Coulter, USA) with CXP Analysis software (Beckman Coulter) using fluorochrome-labeled monoclonal antibodies (CD45 FITC/CD4 PE/CD8 ECD/CD3 PC5, CD19PC7, CD3 FITC/CD(16+56) PE, CD45 PC5, CD5 FITC/CD23 PE/CD19 ECD, CD27PC7, purchased from Beckman Coulter, USA) and Versalyse wash-free lysis (Beckman Coulter, USA).

### Statistical and bioinformatic analysis

The results of 16S rRNA gene sequencing were independently evaluated by two different computer tools. First tool: metagenomics 16S rRNA Workflow MiSeq Reporter Package, provided together with Illumina sequencing platform with applied the GreenGenes database [27]. Second tool: DADA2 pipeline [28] and 16S SILVA database [29] was applied to predict the taxonomic annotation using QIIME2 [30]. Due to the compositional type of such data (CoDa) to WGS data analysis evaluation required CoDa analysis approaches [31] such as Aitchison distance [32, 33] with the aid DEICODE [34]. The sequence quality filtration for the WGS metagenomic data was performed by means of the "metaWRAP read_qc" module [35]. To obtain taxonomic compositions for metagenomic WGS data, we used MetaPhlAn2 [36, 37]. CoDa approaches (Aitchison distance) and non-metric multidimensional scaling (NMDS) were used for bi-dimensional visualization. A balance dendrogram (CoDa dendrogram) was used for building a model of ecological succession of recipient gut microbiota due to FMT. This dendrogram-like graph shows: (a) the way of grouping parts of the compositional vector; (b) the explanatory role of each sub-composition generated in the partition process; (c) the decomposition of the total variance into balance components associated with each binary partition [38, 39]. Before the analysis, removal of rare taxa and substitution of zeros by Bayesian estimation of (non-zero) proportions were performed [40]. For additional analysis unweighted UniFrac distance and Bray-Curtis dissimilarity was used. Visualization was performed in the R statistical environment using vegan package [41] and ggplot2 library (https://ggplot2.tidyverse.org).

To trace distinct donor-derived strains in the recipient metagenomic data we used genome-resolved metagenomic (GRM) approaches based on metagenome-assembled genomes (MAGs). To assemble the MAGs, individual samples from donor and each recipient were used separately. The metaWRAP pipeline was used for the MAGs assembly [35] (contain MEGAHIT [19], CONCOCT [42], MetaBAT2 [43], MaxBin2 [44], and BWA [45]), with the following parameters of resulting bins: completeness >70%, contamination <10%, nucleotide length >2,000,000 bp. Multiple alignments for 43 marker MAGs segments (amino acid sequences), plotting a phylogenetic tree, and subsequent taxonomic annotation was performed by means of CheckM [46].

The MAGs were clustered by alignments, guided by 100% amino acid (AA) similarity between the studied sequences (dist.alignment from seqinr package for R [47]). The clusters obtained were then additionally compared (using full MAGs sequences) by their nucleotide similarity using OrthoANI [48]. To follow the dynamics for donor MAGs in metagenomic samples from recipients, the Anvi’o framework [49] (including Prodigal tool [50]), and Bowtie2 [45] was applied, suggesting a design of contig database from the donor-derived MAGs, alignment of metagenomic samples, as well as visualization of the resulting data. Additionally, for tracking donor-derived bacteria in recipient metagenomes metaSNV [51] profiling based on mOTUs2 pipeline database [52] was used (major allele distance was applied as dissimilarity metric).

## Results

### Clinical observations

**Fig. 2:**
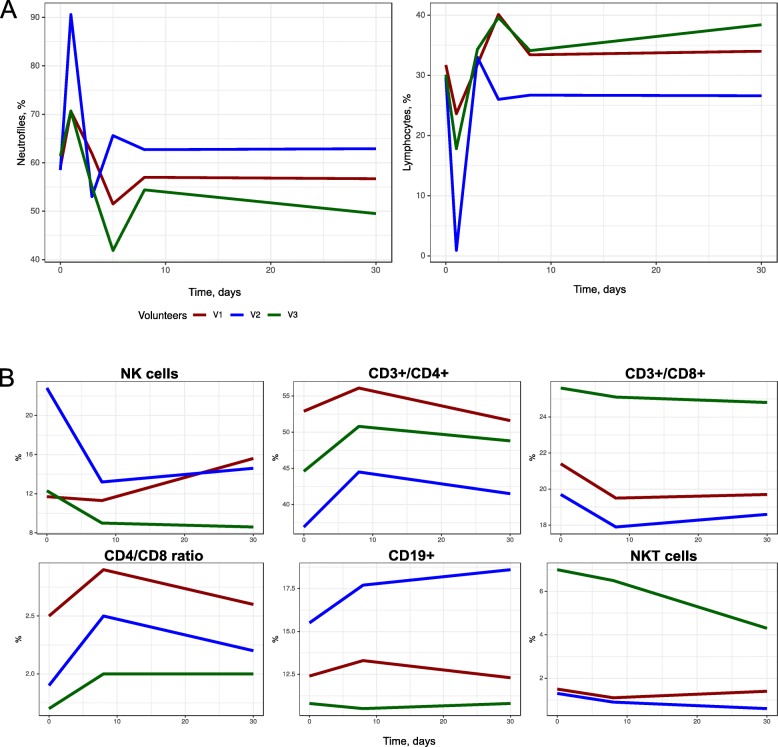
Dynamics of neutrophil counts, lymphocyte counts (**a**) and lymphocyte sub-populations after FMT (**b**). The second volunteer (V2) developed SIRS

Clinical observations of the volunteers were performed during the first month post-FMT. The AEs were registered for all the subjects 8 to 10 hours after taking the capsules (Additional file 6: Table S2). There was no emerging AE past the first 24 hours. V1 and V3 exhibited only grade 1 gastrointestinal AEs post-FMT. The second volunteer (V2) developed a SIRS. Dynamic monitoring of clinical blood cell counts, biochemical blood analysis, and evaluation of lymphocyte subpopulations are presented in Fig. 2. On day 2 of treatment, all laboratory tests were in the normal range, except for increased blood neutrophil counts from 59.1% (5.1 ×10^9^/l) to 70.6% (8.9 ×10^9^/l), and from 61.4% (6.3 ×10^9^/l) to 70.7% (6.9 ×10^9^/l), for V1 and V2, respectively. Blood lymphocyte counts showed a decrease from 31.7% to 23.6%, at similar absolute lymphocyte numbers (2.8 ×10^9^/l and 3.0 ×10^9^/l). V3 exhibited a relative decrease of lymphocyte counts, both in percentage (30.1% to 17.8%) and in absolute values (2.8 ×10^9^/l to 1.7 ×10^9^/l). A limited number of immune parameters included counts of total leukocytes and their main subpopulations. Gross changes in total leukocyte and neutrophil counts were observed in V1 and V2. Meanwhile, the CD3+/CD4+ and CD4+/CD8+ ratios seemed to increase by day +8 after FMT, with a rapid reversal to normal values. These changes do not mean any prolonged immune depression, as compared, e.g., to leukopenia observed following cytostatic therapy. Rather, they resemble a systemic acute response to antigenic stimulation.

For V2, we observed a number of pronounced symptoms, which required additional therapy. Ciprofloxacin was administered at a daily dose of 500 mg for 3 days, and the 2nd round of FMT in this subject was canceled. V2 developed a clinical pattern of the systemic inflammatory response (fever, with the one-time rise of body temperature to 39.1 ^∘^C, with shivers and tachycardia of 102 per minute on the day after administration). The blood changes corresponded to acute bacterial infection: leukocytosis to 16.7 ×10^9^/l, neutrophils 90.6% (15.1 ×10^9^/l), absolute lymphopenia (0.9%, 0.2 ×10^9^/l). Blood smear counts showed an increase in band forms, 10% (1.67 ×10^9^/l), segmented forms, 80% (13.36 ×10^9^/l); toxic granulation in neutrophils and decrease of lymphocytes, 4% (0.66 ×10^9^/l). C-reactive protein levels were within normal ranges, a marginal increase of *γ*-glutamyl transpeptidase to 56.7 U/l (normal range: 0-55 U/l) and ALT to 62 U/l (normal range: 0-50 U/l) was noted on day +2. Clinical chemistry parameters of V1 and V3 were within normal ranges during the treatment course.

The lymphocyte subpopulations were examined before FMT, as well as on day +9 and day +30. By day +9, an increased percentage and absolute numbers were observed for T-helpers CD3+CD4+, CD19+CD23+ cells; CD4/CD8 ratio; as well as a decrease in lymphocyte subpopulations, i.e., T-cytotoxic CD3+CD8+ lymphocytes, and NK cells (CD3-CD16+56+). By day 30, a reverse dynamics to normal values was revealed. The number of recipients was insufficient to evaluate the statistical significance of the observed changes. However, we could be assumed an association between adverse effects and immune system perturbation.

### Gut microbiome changes after FMT

**Fig. 3:**
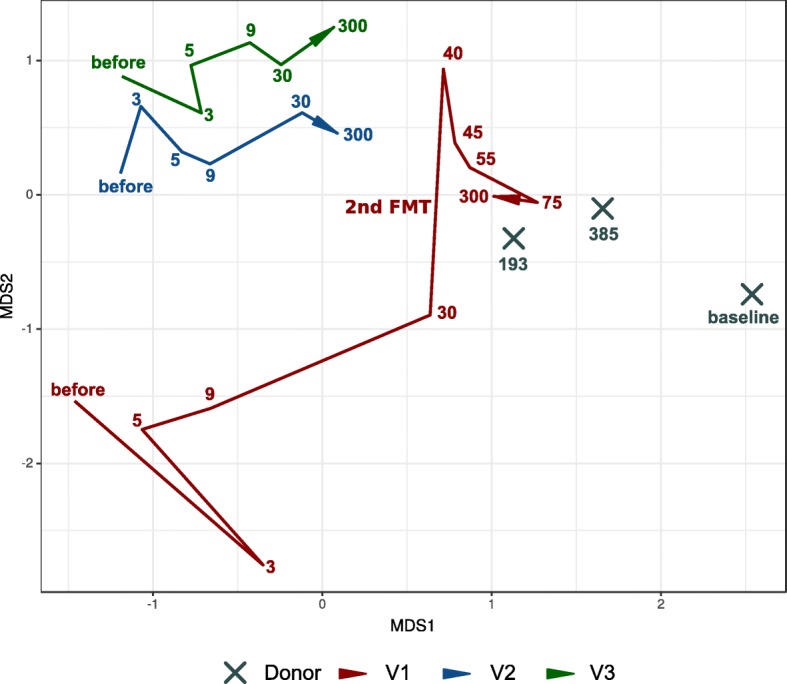
Movement of recipient samples to the donor during the observation time based on 16S rRNA gene sequencing taxonomic composition. Bi-dimensional plot obtained by Aitchison distance with the aid of DEICODE. Donor samples: X. Volunteer’s samples: red / blue / green colors (see figure legend). The lines denote the evolution of the volunteer’s samples in time (different time points). The days after FMT procedure (or baseline for donor samples) denoted by color numbers

16S rRNA gene sequencing (16S seq) data analysis was performed in two independent laboratories and the results were consistent in both assays. Summary sequencing statistics are presented in Additional file 6: Table S3. The NMDS bi-dimensional plot obtained with using Aitchison distance and 16S seq taxonomic data is presented in Fig. 3. It shows the convergence of the recipients’ gut taxonomic profiles to the donor profile within 300 days after the FMT. Interestingly, the gut metagenomic profile of V1 showed a dramatic change after the second FMT round procedure from the same donor (2 days after the second FMT round). However, further samples showed a return to the donor pattern. Additionally, analysis using NMDS and unweighted UniFrac distance confirmed previous results (see Additional file 1: Figure S1A).

**Fig. 4:**
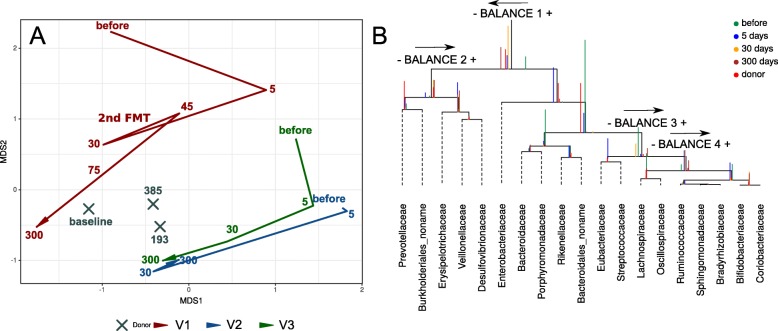
Shifts of the taxonomic profile of microbiota in volunteers towards donor values over the observation time. The figure is based on shotgun sequencing data. **a** on-metric multidimensional scaling bi-dimensional plot of MetaPhlAn2 taxonomic profile (genera level relative abundances), based on the Aitchison distance. The lines denote the evolution of the volunteer’s samples in time (different time points). The days after FMT procedure (or baseline for donor samples) denoted by numbers. **b** CoDa dendrogram which characterizes association of bacterial families, balances presented as edges. Decomposition of total variance by balances between groups of families is shown by vertical bars. Mean values of balances is shown by anchoring points of vertical bars. Color of vertical bars corresponds to time points. Color rectangles highlighted families belonging to important balances. The arrows direction indicates the predominance of this balance part in the donor. MOTUs with no family information are collapsed into the no-name family

Shotgun metagenomic sequencing was another method for studying changes in the intestinal microbiota profile of the recipients, which yielded 23.1 ± 3.7 M of 250 bp reads per sample (98.3 Gbp in total) after quality control. Seventeen metagenomic samples were sequenced with the shotgun method (6 for the V1, 4 for the V2 and V3 and 3 samples for the donor). The sequencing summary statistic is presented in Additional file 6: Table S3. A total of 74 genera were detected in all samples. The dataset of relative abundances of bacterial genera is shown in Additional file 6: Table S4. The shotgun sequencing confirmed the 16S seq data with a similar pattern of changes towards the donor profile (Fig. 4a). Similar results were obtained by NMDS bi-dimensional visualization using Bray-Curtis dissimilarity (see Additional file 1: Figure S1B).

For constructing the model of microbiota succession caused by FMT, the balance dendrogram (CoDa dendrogram) was used. This approach allows identifying specific balances (ratio between taxonomic abundances) that are involved in the reshaping of the microbiome of recipients [31, 38]. This model describes the intensity of taxonomic reshapes when moving the recipients’ profiles to the donor-specific parameters (see Fig. 4b). Immediately, on the fifth day after FMT, the recipients relatively increased the content of *Prevotellaceae*, unknown *Burkholderiales*, *Erysipelotrichaceae*, *Vellonellaceae*, and *Desulfovibrionaceae*; however, the shift towards *Prevotellaceae*, unknown *Burkholderiales*, was more pronounced at day 5. At the same time, the relative increase of *Lachnospiraceae*, *Oscillospiraceae*, *Rumminococaceae*, *Sphingomonadaceae*, *Bradyrhizobiaceae*, *Bifidobacteriaceae* and *Coriobacteriaceae* occurred less quickly and more smoothly. Also, the relative abundance of *Enterobacteriaceae*, *Bacteroidaceae*, *Porphyromonadaceae*, *Rikenellaceae*, unknown *Bacteroidales*, *Eubacteriaceae*, and *Streptococcaceae* decreased gradually towards the donor-like profile.

Altogether, the obtained results show a directed change in the gut microbiota composition of volunteers, namely pre-FMT profiles of the recipient microbiota have changed after FMT and become similar to the donor microbiota.

### Identification of donor bacteria in the recipient metagenomes

**Fig. 5:**
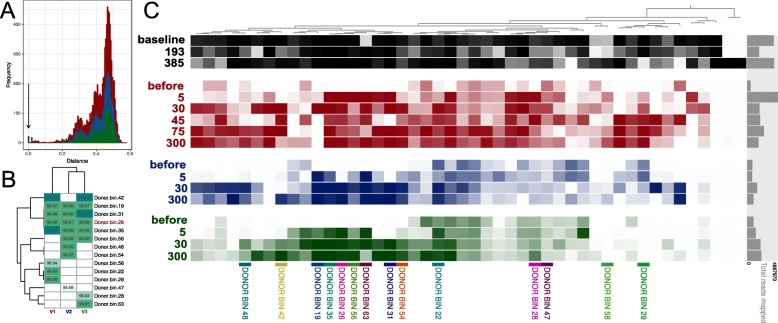
Comparison of similarity between donor and recipients metagenome-assembled genomes (MAGs). **a** The AA distance based on 43 marker proteins between all donor MAGs and all MAGs of all recipients. Arrow shows that some MAGs in donor and recipient is present with absolute similarity of marker genes sequence. **b** The average nucleotide identity (ANI) between similar donor and recipients MAGs. The MAGs with 100% AA similarity of 43 marker proteins were selected. **c** Anvi’o plot denoted prevalence of donor MAGs across all metagenomic samples. Detection value (proportion of nucleotides in a contig that are covered at least 1x (according to http://merenlab.org/2017/05/08/anvio-views) was used as an abundance metric, which is shown as color brightness. Black color denotes detection value of donor MAGs in the donor samples, red – in the V1 samples, blue – in the V2 samples, green – in the V3 samples. DONOR BIN – clusters of metagenome-assembled genomes similar to the donor bacteria. The days after FMT denoted by numbers. The mapping of recipient metagenomic reads to donor MAGs was performed with 100% similarity

Taxonomic profiling methods may reveal general changes of the taxonomic profile for the gut microbiota. However, it is important to examine the engraftment of the donor bacteria in recipients. To assess the engraftment of the donor bacteria using the obtained shotgun sequencing data, we used genome-resolved metagenomics – an approach allowing to restore bacterial genome from the metagenomic data (metagenome-assembled genomes – MAGs). This method is based on the metagenomic assembly and clustering of contigs through a metagenomic binning procedure and others specific manipulations (see “Methods” section). As a result, 243 MAGs were assembled for all metagenomic samples both from donor and recipients. For the donor 46 MAGs were obtained, for each of the volunteers 87, 56, and 54 MAGs, respectively (note that these MAGs represent microbes from both pre- and post-FMT time points). Further, based on 43 marker single-copy proteins, the place at the dendrogram for each MAG was determined (see Additional file 2: Figure S2), and appropriate taxonomic annotation was ascribed with the CheckM tool. We detected 14 donor-like MAGs in which 100% amino acid similarity of marker proteins was observed (see Fig. 5a). The changes in the relative abundance of these 14 MAGs are shown in Additional file 3: Figure S3. The similarity of the nucleotide sequence (average nucleotide identity – ANI) between donor and recipient MAGs was also high (see Fig. 5b). Anvi’o visualization for the mapping results of the reads from recipient samples in the donor MAGs is shown in Fig. 5c.

DONOR_BIN_26 didn’t show the 100% amino acid homology with MAG of V1 and V3. However, they were similar in their nucleotide composition. These discordances could be explained by some metagenomic assembly artefacts and binning, thus resulting in chimeric contigs. The given approach shows a rather big number of false-negative results; however, it allows to detect successful cases from nonspecific findings. Of 14 donor MAGs with complete amino acid sequence similarity in marker proteins, 10 may be considered as successfully engrafted in at least one recipient. The DONOR_BIN_28, DONOR_BIN_47, DONOR_BIN_22, DONOR_BIN_22, DONOR_BIN_22 did not enter this list due to the following reasons: (1) nucleotide identity from recipient MAGs (threshold <99.90% ANI); (2) they were covered by reads after FMT (not 100% certainty of their donor origin). By taxonomic annotation, these "strong" colonizers belong to the following orders: *Bacteroidales* (n=5), *Clostridiales* (n=3), *Selenomonadales* (n=1). Many donor MAGs didn’t show 100% similarity with recipients MAGs in the two parameters described above. However, this MAGs appeared in the recipients after FMT. This can be explained by the chimeric contigs and/or sequencing errors when assembling recipients MAGs. In addition, similar recipient bacteria can increase after FMT.

Ten MAG clusters, similar in amino acid sequences of marker proteins, were exclusively present in the recipient metagenomes (see Additional file 3: Figure S3). Based on the criteria of an nucleotide similarity (ANI >99.90%), there is evidence that the observed changes of several genera of bacteria abundances were donor-independent. Either these expanding bacteria were in donor samples, but were not found due to insufficient read coverage, or the FMT procedure induces the relative expansion of certain types of recipient bacteria (for example, see Additional file 3: Figure S3A). We have also revealed 4 cases of similar MAG sets in V2 and V3 that decreased relatively after FMT in both recipients. V2 and V3 have similar patterns of decrease and increase of some similar MAGs (see Additional file 3: Figures S3 D, F, H, I, J and Additional file 4: Figure S4). More detailed information about MAGs assembly is presented in Additional file 6: Table S5.

Additionally, SNV-profiling using mOTUs2 pipeline and metaSNV was performed. We detected that after FMT the number of mOTUs identical to donor were increased. The results are present in Additional file 5: Figure S5.

## Discussion

FMT has been increasingly used for the treatment of different disorders. The majority of studies have focused on the evaluation of FMT consequences in patients with rCDI, ulcerative colitis, and Crohn’s disease. It was speculated that in these diseases the therapeutic effect is based on the expansion of the donor flora and on the correction of defects in the species composition [53–55]. The correction of the intestinal microbiome leads to the restoration of short-chain fatty acids and bile acid metabolism, altered immune response, the profile of cytokines and chemokines, and augmentation of intestinal wall reparation. The mentioned processes may be immediately or indirectly affected by other medications, e.g. granulocytic colony-forming factor, glucocorticosteroids used in hematopoietic stem cell transplantation, antibiotics used in pseudomembranous colitis, aminosalicylates, anti-TNF monoclonal antibodies in Crohn’s disease, and nonspecific ulcerative colitis. While the changes in microbiome after FMT are well established, it is unclear whether they can be attributed only to FTM, as more complex mechanisms may be involved. Therefore, the study of FMT effects in healthy volunteers is important to understand the mechanisms behind the efficacy of this procedure and potentially to improve its outcome for patients (e.g. by rules of donor selection).

The present study resulted in several important findings. First, we demonstrated that FMT even with a small stool mass (11-22 g) induces profound changes in healthy persons with normal microbiota composition. The convergence of the recipient taxonomic composition to the donor-like state following the FMT procedure was demonstrated for various diseases [55–58], but comparable changes were observed in the healthy volunteers. Thus, the effects of FMT are comparable in the normal and pathological conditions, indicating that the replacement of the missing bacterial populations is not a unique event for disease conditions. Second, we observed that the composition of the gut flora is altered due to engraftment of donor bacteria. Although a sequencing artefact cannot be excluded, all published studies with FMT indicate significant increase in the overall bacterial diversity, which is not only related to the growth of the donor flora [59, 60]. The potential mechanisms behind the activation of recipient flora might be horizontal gene transfer [61], effects of the non-bacterial stool components [62], and functional interactions between microbial communities [63]. Further studies are required to elucidate the exact mechanism(s). However, we have identified some features of the restructuring process. *Prevotellaceae* and unknown *Burkholderiales* colonize faster than others. This might be a "hub" bacteria that allow to develop a new "version" of the recipient gut community, which would include recipient-derived and donor-derived characteristics.

The number of potential applications of FMT is exponentially growing: decolonization from antibiotic-resistant bacteria prior to stem cell transplantation [60], modulation of response to cancer immunotherapies, like anti-PD-1 antibodies [64], vaccination against respiratory pathogens [62], amelioration or prevention of non-gastrointestinal infections, like malaria [65], treatment of autism [43] and depression [66]. However, there is no evidence that FMT induces long term changes in subjects with no previous damage to microbiota due to antibiotics treatment or to the underlying condition. This study provides the first proof of principle that, even in a healthy person, the procedure induces long-term changes with a shift towards donor profile. Here we show that FTM induced several gastrointestinal adverse events and early inflammatory response at day 2 in V1 and V2 cases, i.e., a shift to band leukocyte forms with normal C-reactive protein levels in healthy recipients. This suggests the development of massive antigenic exposure with leukocytosis where, even in the absence of detectable evidence, the development of septic state requiring antibiotic treatment cannot be excluded. Indeed, a SIRS-like syndrome was diagnosed in one of our cases (V2), thus requiring urgent antibiotic therapy. It should be regarded as a serious adverse effect of FMT occurring in an obviously healthy immunocompetent person [23]. The adverse effects after FMT are classified as mild, serious and severe. Lethal outcomes following FMT are accidental and are most likely associated with underlying disease, or exacerbating comorbidities. A recent detailed review of the side effects in FMT was based on data obtained from 1998 to 2015 [67]. Severe AEs were mostly related to endoscopic procedures and aspiration. The use of capsule FMT seems to minimize the risk of the procedure. The observed mild adverse effects may require correction using only symptomatic therapy, like anti-inflammatory and spasmolytic drugs. Leukocytosis and neutrophilia, along with relative and absolute lymphopenia may be a near-normal variant following FMT. One volunteer did receive a short treatment of ciprofloxacin for systemic inflammatory response syndrome (SIRS), but the sequencing data still indicated the shift towards the donor pattern of the microbiota. In the previous study, though, early antibiotic use was reported to compromise the efficacy of the procedure [68].

Although the study group was small, the observed changes were consistent with previous preclinical studies [69, 70] and case reports [71]. There was a transient decrease in total lymphocyte count, a decrease of CD8+ cells, decrease of NK cells and increased CD4/CD8 ratio. The effect was most prominent in the volunteer with SIRS. The downregulation of lymphocyte response might have comparable mechanisms to the one induced under bacterial septic conditions [72]. This AE might be one of the mechanisms behind the FMT benefit in autoimmune disorders.

In the clinical studies it was demonstrated that the efficacy of FMT was based on such genera as *Ruminococcaceae*, *Lachnospiraceae* and *Prevotellaceae* [59, 73]. These were the bacterial species that were consistently expanded in the volunteer samples. It is unclear if they directly drive the therapeutic effect of the FMT or are just a marker of the changes that induce the response. The question regarding the optimal donor and driving force for each indication is still open. Only accumulation of clinical observations will give the answer to this intriguing question. Another approach used by the industry in the early clinical trials is the mixture of products from a large number of donors (https://clinicaltrials.gov/ct2/show/NCT03497806), but the benefits and drawbacks of such an approach are still to be evaluated.

Our study, though rather limited, shows some potential risks of the allogeneic FMT procedure, even when performed in healthy volunteers. Appropriate studies in healthy persons should be performed cautiously under strict medical supervision. The FMT procedure should be administered only if the expected benefits sufficiently prevail over the possible risks. Moreover, the FMT protocol should be detailed and reproducible to obtain stable clinical outcomes.

## Conclusions

The main conclusion of the present study is the confirmation of the long-term microbiota composition conversion by FMT in healthy subjects. The microbiome composition in recipients shifted towards the donor profile. The most important finding was the relative expansion of donor-derived bacteria inside healthy recipients gut. Additional important findings may be certain rules of community succession after FMT. Perhaps in the future, the description of these rules will allow the microbiota to be controlled and directed from one state to another. However, researchers and physicians should take a responsible approach to the FMT procedure and apply this procedure only when the positive effects far exceed the possible risks, since fecal transplantation may be associated with severe side effects.

## Supplementary information

**Additional file 1** Additional file 1: Figure S1 Non-metric multidimensional scaling bi-dimensional plots of MetaPhlAn2 taxonomic profile (genera level relative abundances), based on the unweighted UniFrac distance (A) and Bray-Curtis dissimilarity (B). The lines denote the evolution of the volunteer’s samples in time (different time points). The days after FMT procedure (or baseline for donor samples) denoted by numbers.

**Additional file 2** Additional file 2: Figure S2 Recipient MAGs with donor MAGs 100% amino acid similarity of 43 marker protein relative abundance change.

**Additional file 3** Additional file 3: Figure S3 Similar recipient MAGs (with 100% similarity of 43 marker proteins) relative abundance change.

**Additional file 4** Additional file 4: Figure S4 The ANI between similar recipient MAGs. Recipient MAGs with 100% AA similarity of 43 marker proteins were selected.

**Additional file 5** Additional file 5: Figure S5 Comparison of similarity donor and recipient bacteria based on metaSNV profiling. **(A)** Histograms shows frequency of distances between major donor sample (used for FMT procedure), additional donor samples and recipient metagenomes. Metagenomic samples obtained before FMT were removed. **(B)** Evolution of distances between major donor sample and metagenomic samples of each recipient over time.

**Additional file 6** Additional file 6: Table S2 Adverse effects after FMT in healthy volunteers (scored by Common Terminology Criteria for Adverse Events (CTCAE) Version 5.0.)
Additional file 6: Table S3 Sequencing and general statistics.
Additional file 6: Table S4 Relative abundance of microbial genera and viruses in the WGS metagenomes.
Additional file 6: Table S5 MAGs assembly and taxonomic annotation statistics.

## Data Availability

We made data obtained in this study publicly available at (http://download.ripcm.com/add_files), where are presented (1) the QIIME2 and MetaPhlAn2 taxonomic tables; (2) the Anvi’o profile to interactively visualize and further investigate donor MAGs mapping profiles across samples; (3) the distribution statistics for each donor MAG across samples; (4) FASTA files for each MAGs. Raw metagenomic data are also deposited at the NCBI Sequence Read Archives under the BioProjects accession numbers PRJNA509769 and PRJNA510036.

## Abbreviations

AA: Amino acid
AE: Adverse effect
ALT: Alanine aminotransferase
ANI: Average nucleotide identity
CMV: Cytomegalovirus
CoDa: Compositional data
ESBL: Extended-spectrum beta-lactamase
FMT: Fecal microbiota transplantation
GRM: Genome-resolved metagenomics
HHV-6: Human betaherpesvirus 6
HIV: Human immunodeficiency virus
HSV-1: Human alphaherpesvirus 1
HSV-2: Human alphaherpesvirus 2
MAG: Metagenome-assembled genome
mOTU: Metagenomic operational taxonomic unit
MRSA: Methicillin-resistant staphylococcus aureus
NK: Natural killer
NMDS: Non-metric multidimensional scaling
rCDI: Recurrent clostridium difficile infection
SIRS: Systemic inflammatory response syndrome
SNV: Single nucleotide variant
VRE: Vancomycin-resistant enterococci
WGS: Whole genome sequencing
