# Supplementary figures and images for: Long-term impact of fecal transplantation in healthy volunteers
Source: BMC Microbiol. 2019 Dec 30;19:312. doi: 10.1186/s12866-019-1689-y (PMC6938016; doi:10.1186/s12866-019-1689-y)

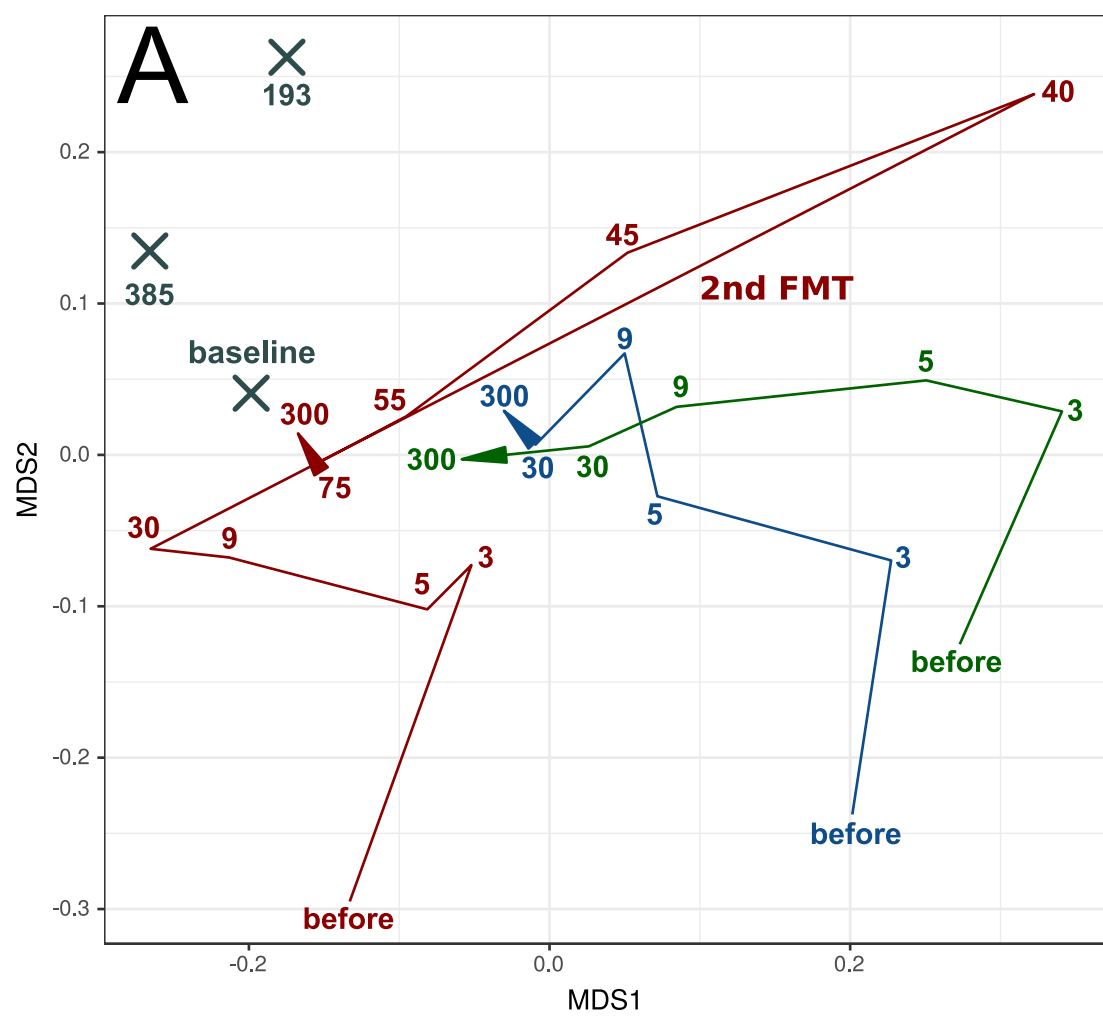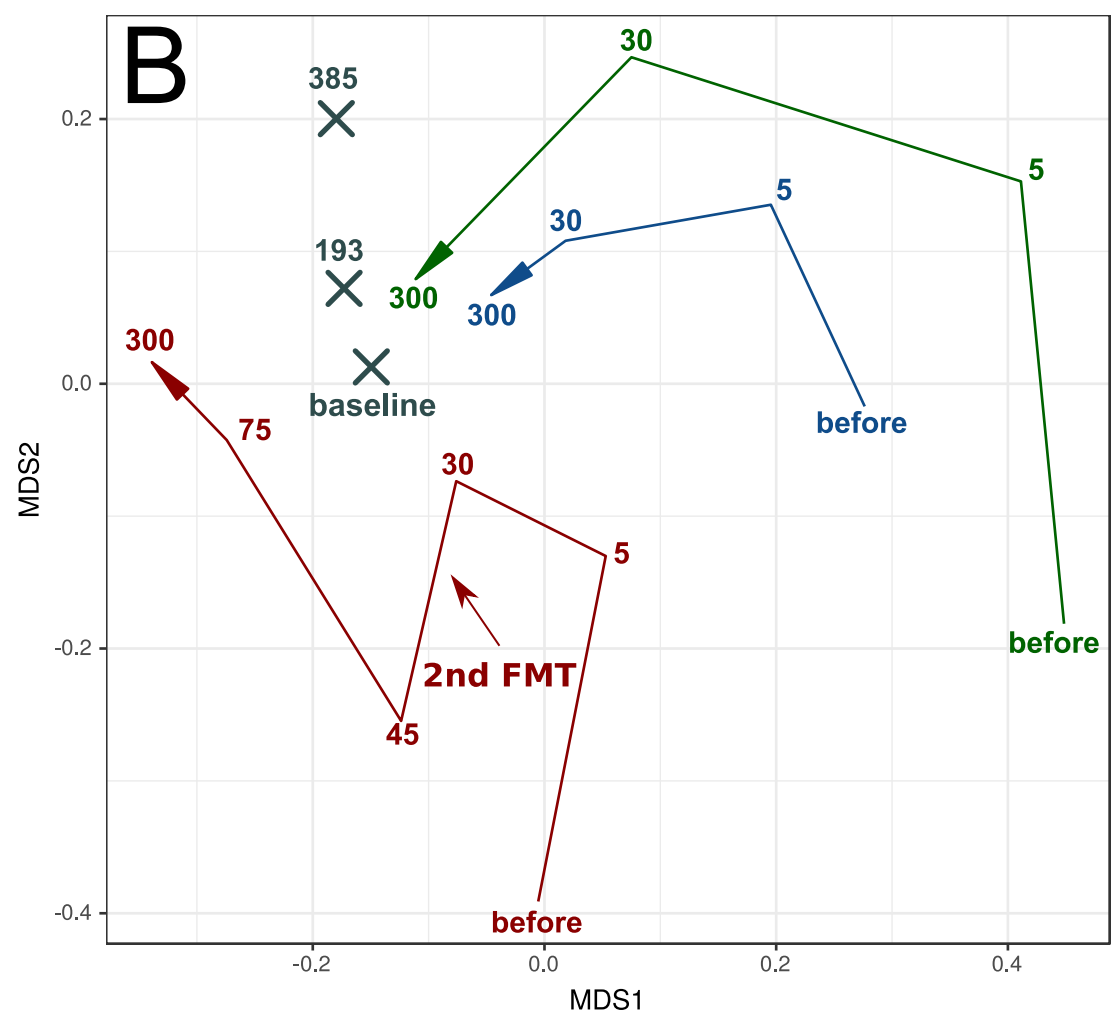

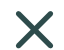 Donor
 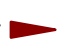 V1
 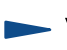 V2
 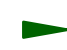 V3

Supplement: Supplementary file 1 — Additional file 1 Additional file 1: Figure S1 Non-metric multidimensional scaling bi-dimensional plots of MetaPhlAn2 taxonomic profile (genera level relative abundances), based on the unweighted UniFrac distance (A) and Bray-Curtis dissimilarity (B). The lines denote the evolution of the volunteer’s samples in time (different time points). The days after FMT procedure (or baseline for donor samples) denoted by numbers. [file 12866_2019_1689_MOESM1_ESM.pdf]

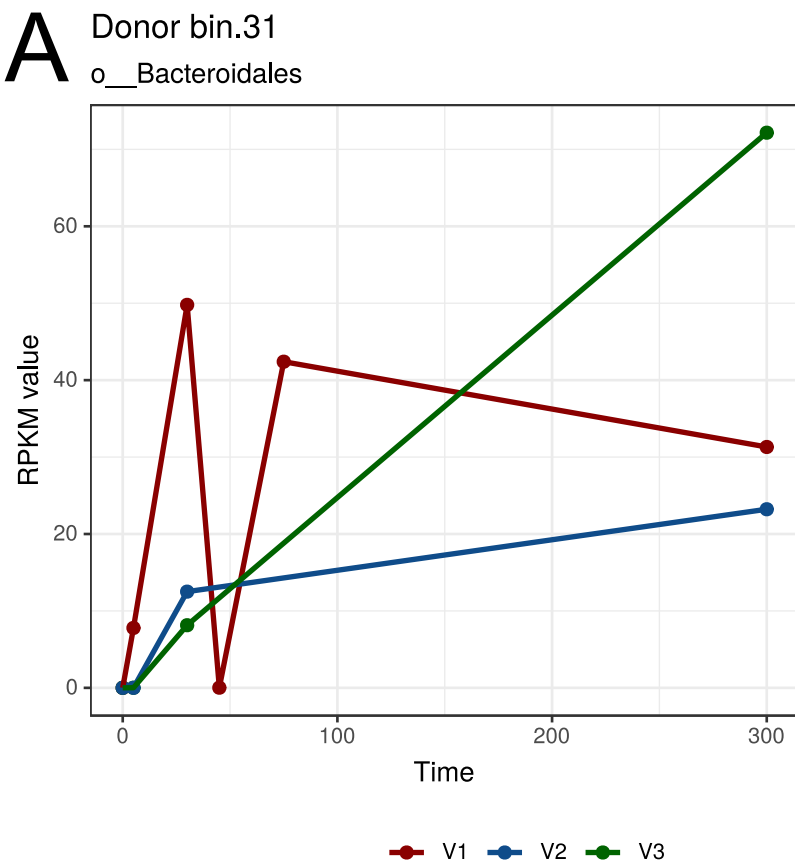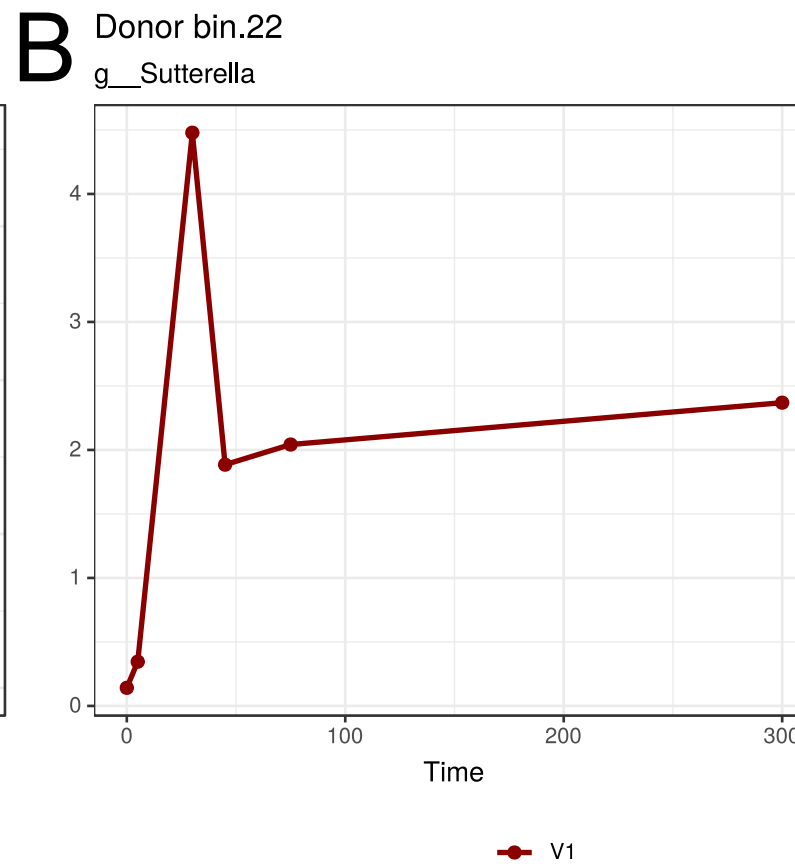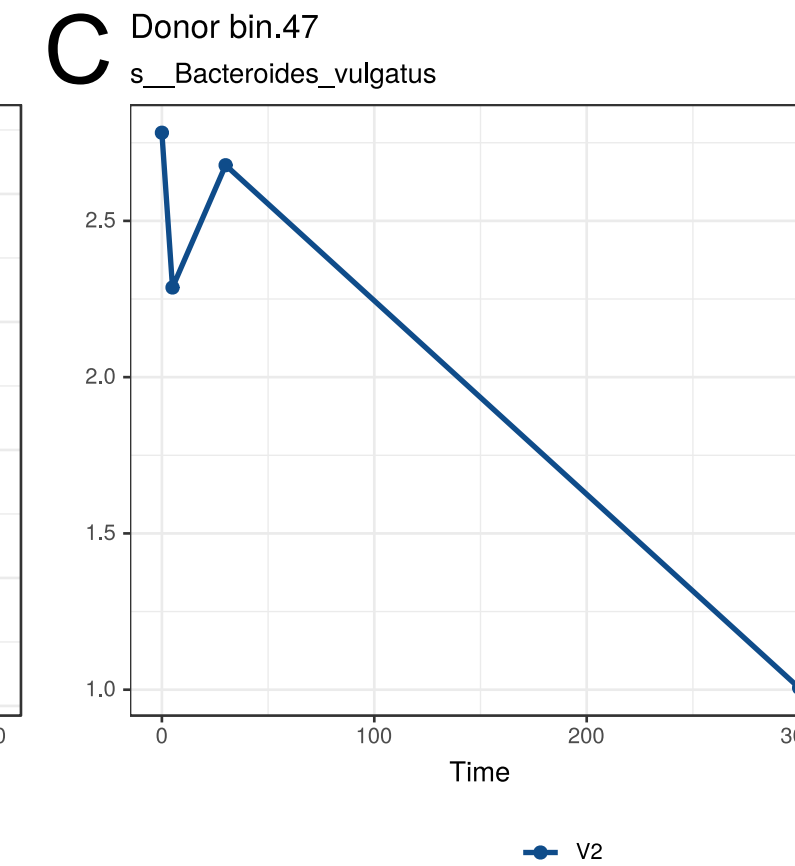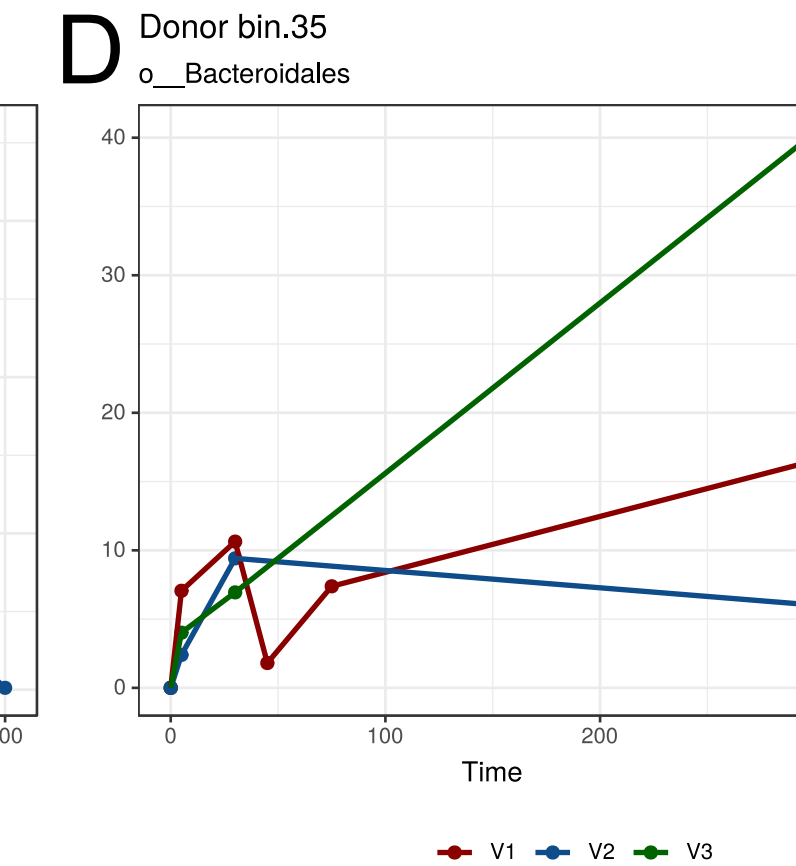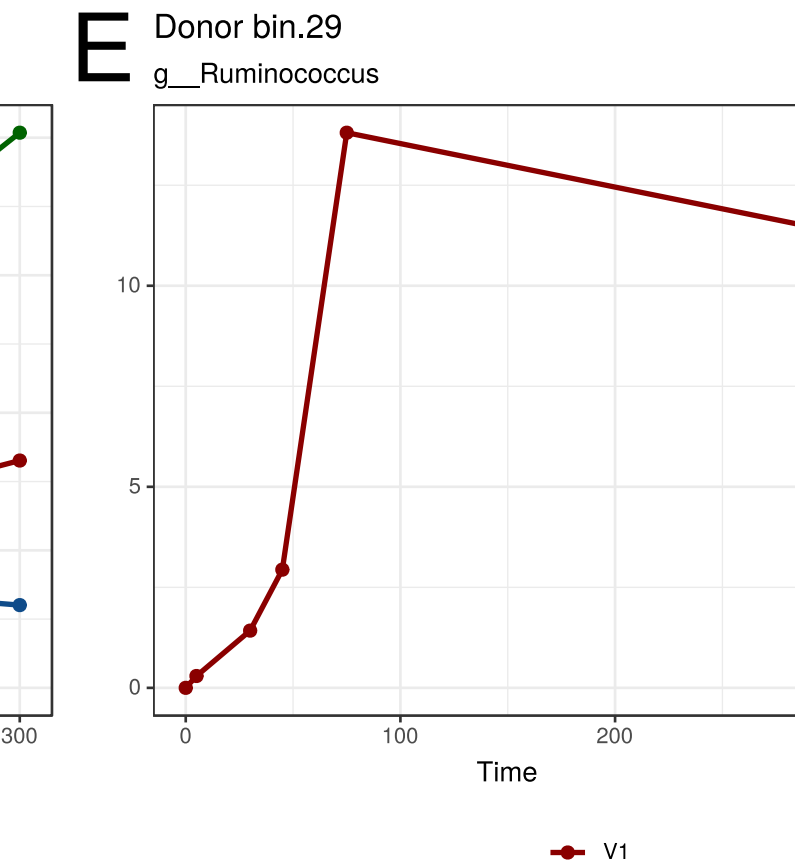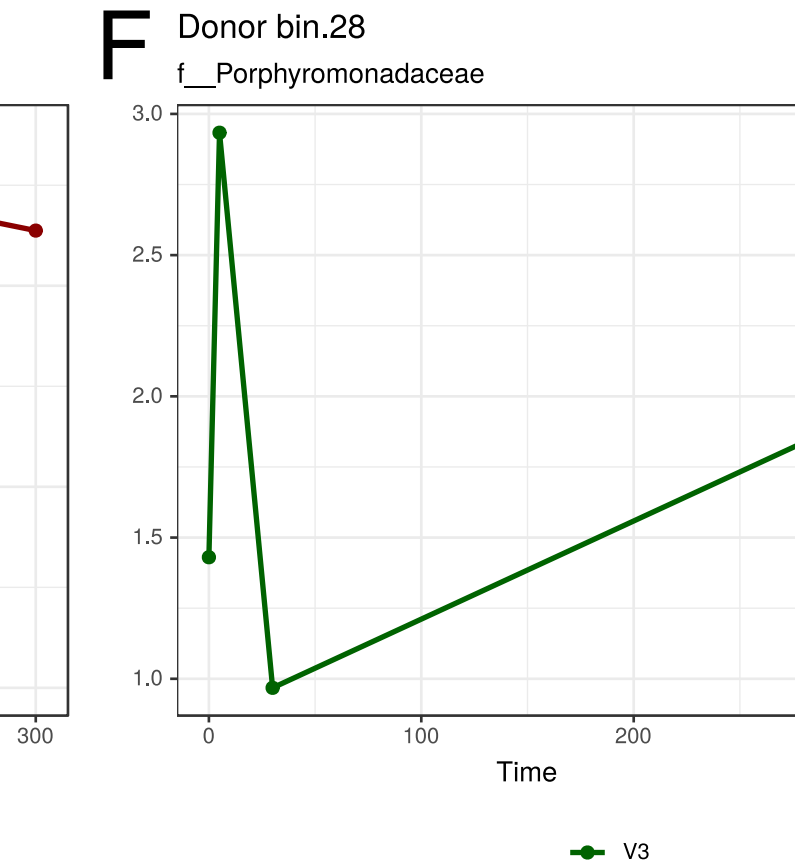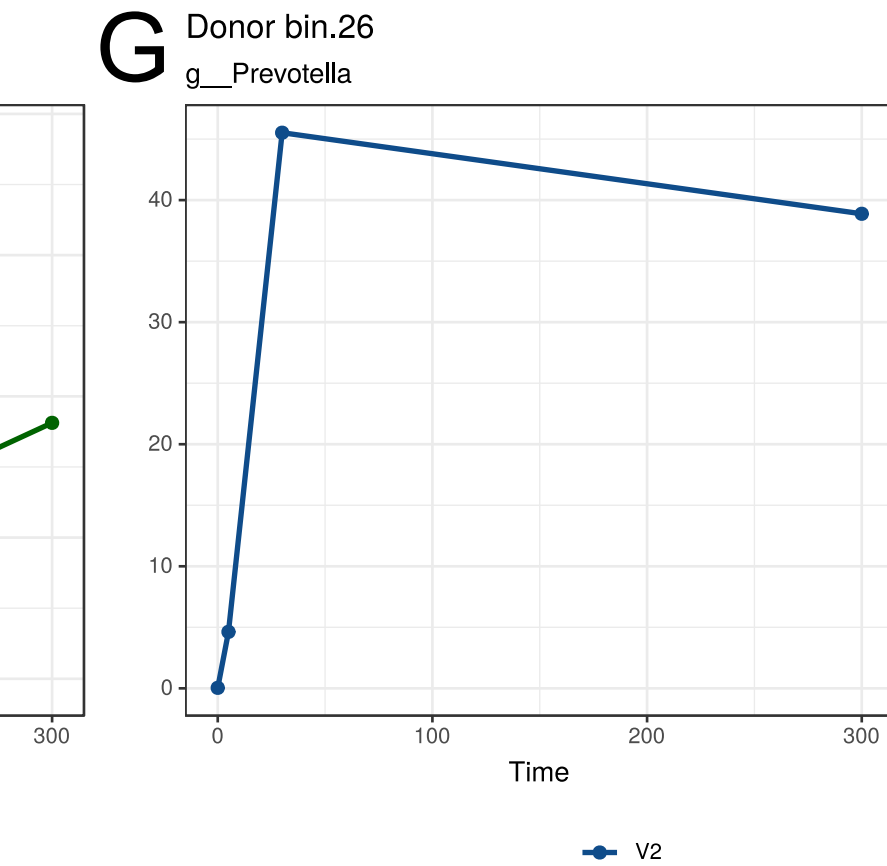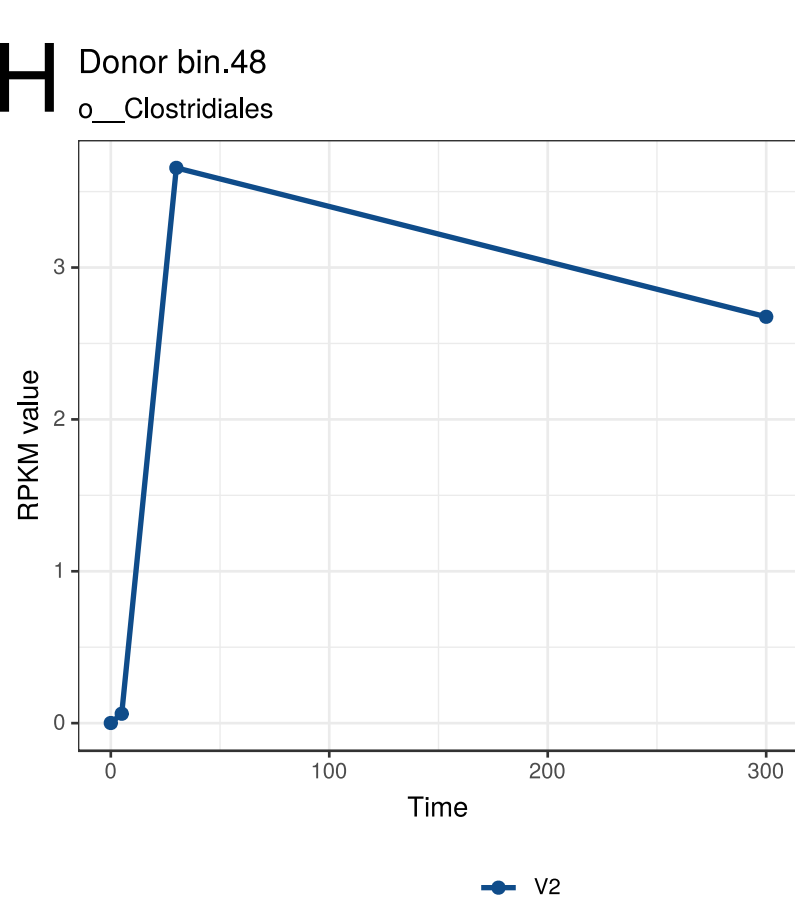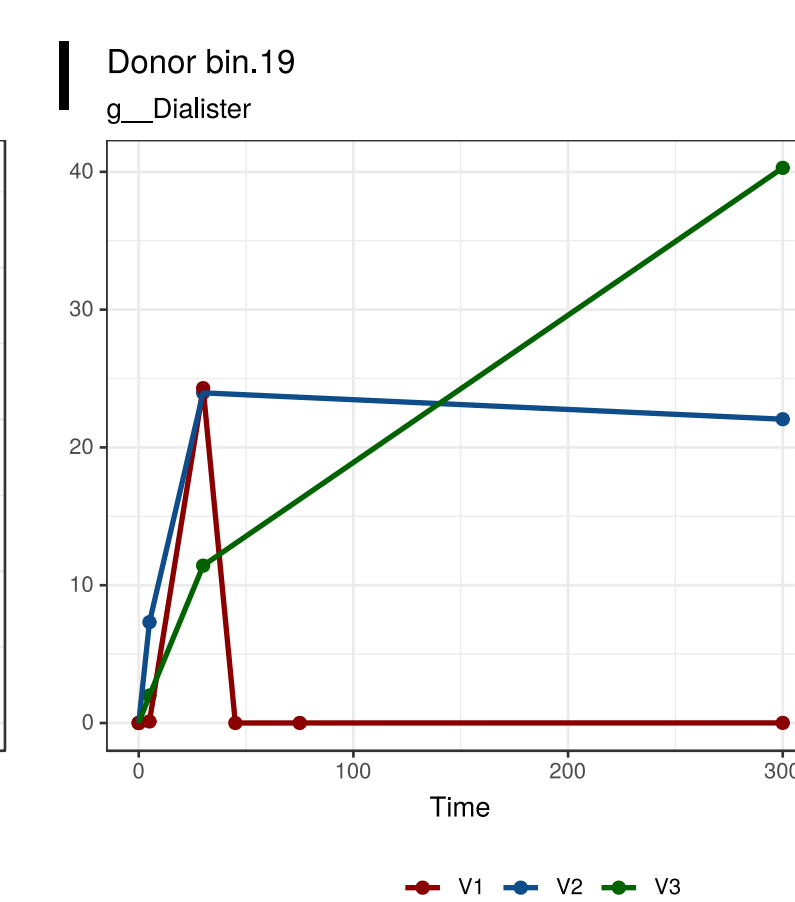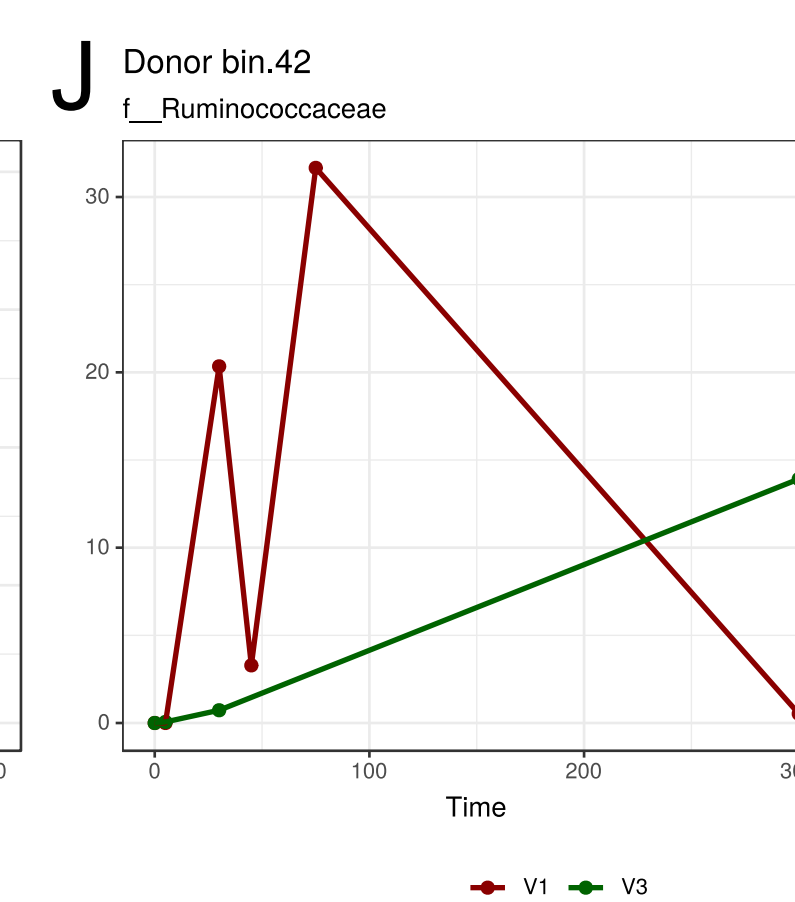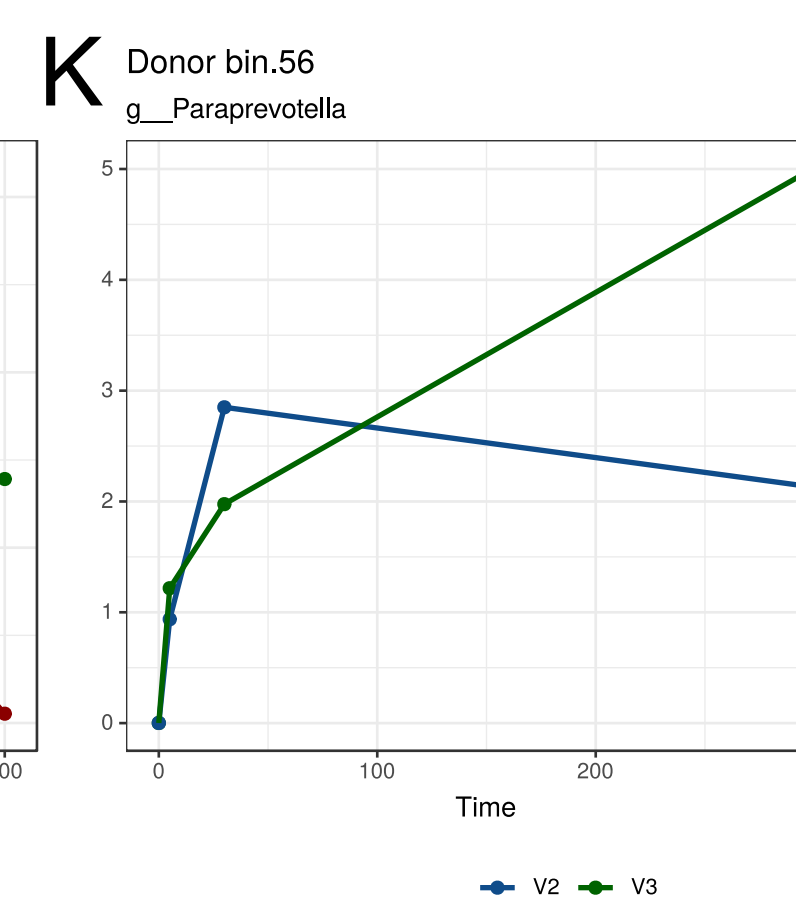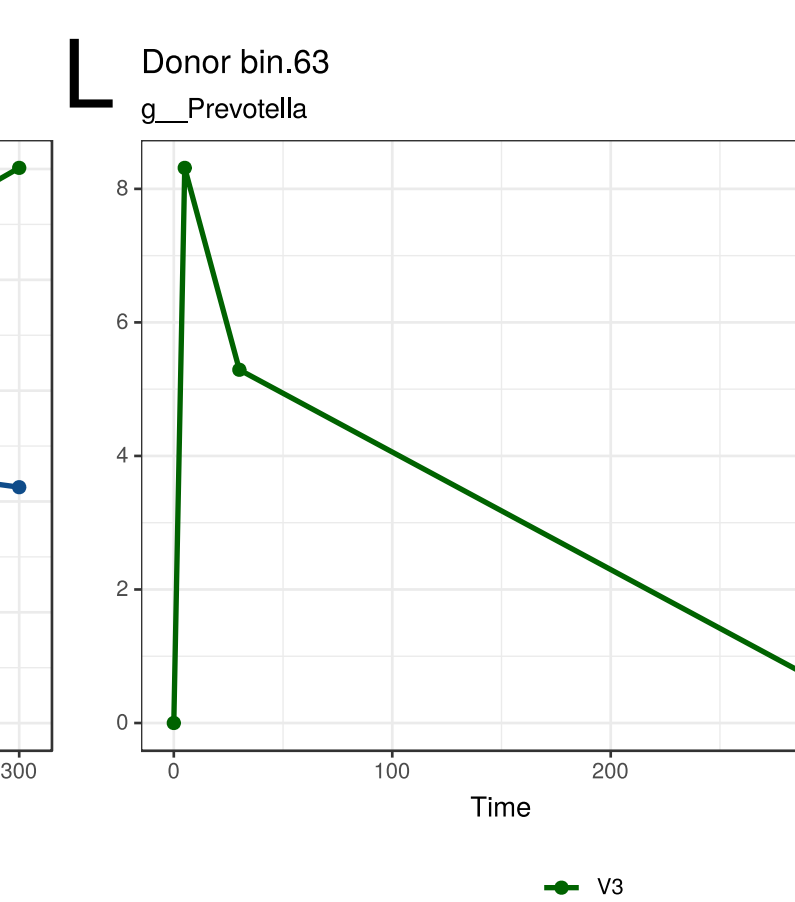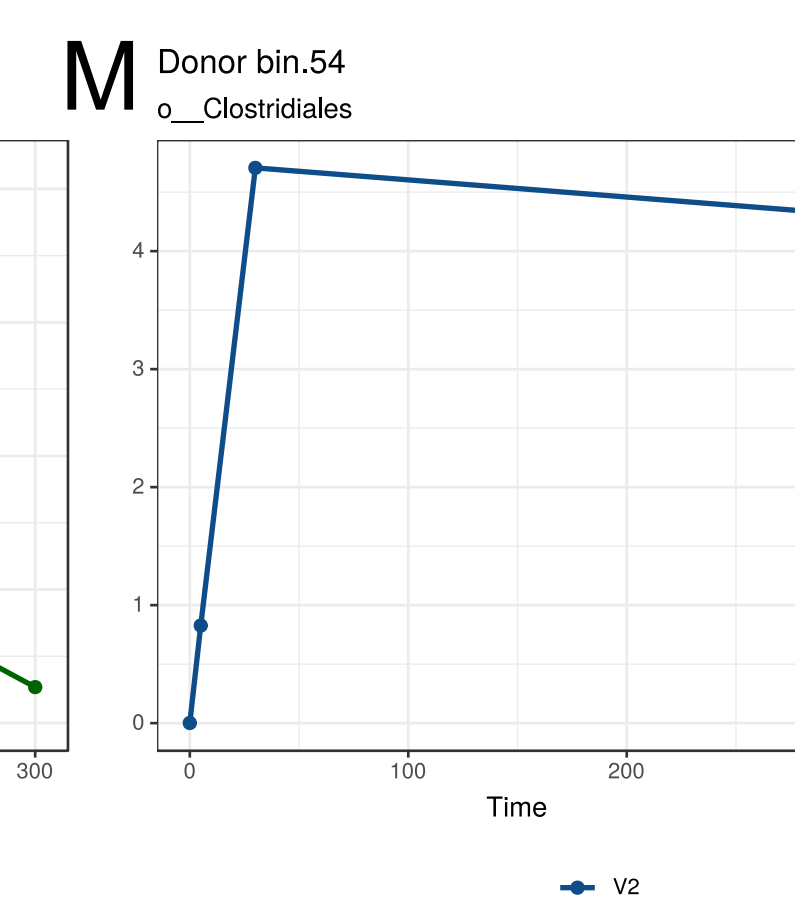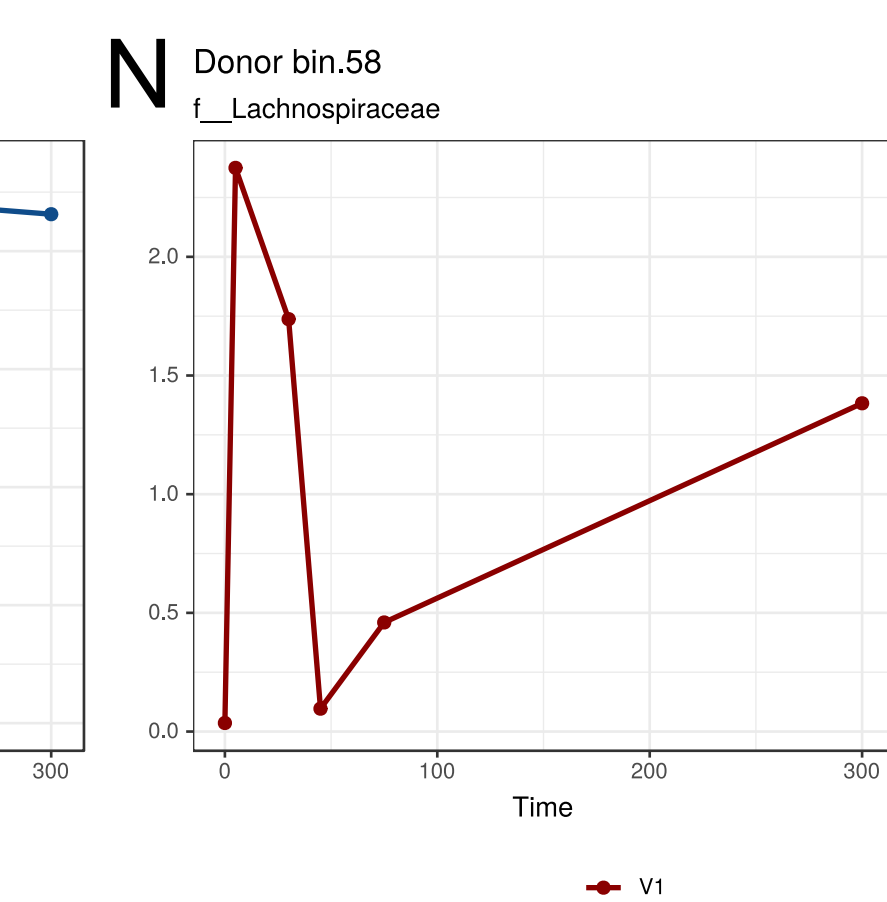

Supplement: Supplementary file 2 — Additional file 2 Additional file 2: Figure S2 Recipient MAGs with donor MAGs 100% amino acid similarity of 43 marker protein relative abundance change. [file 12866_2019_1689_MOESM2_ESM.pdf]

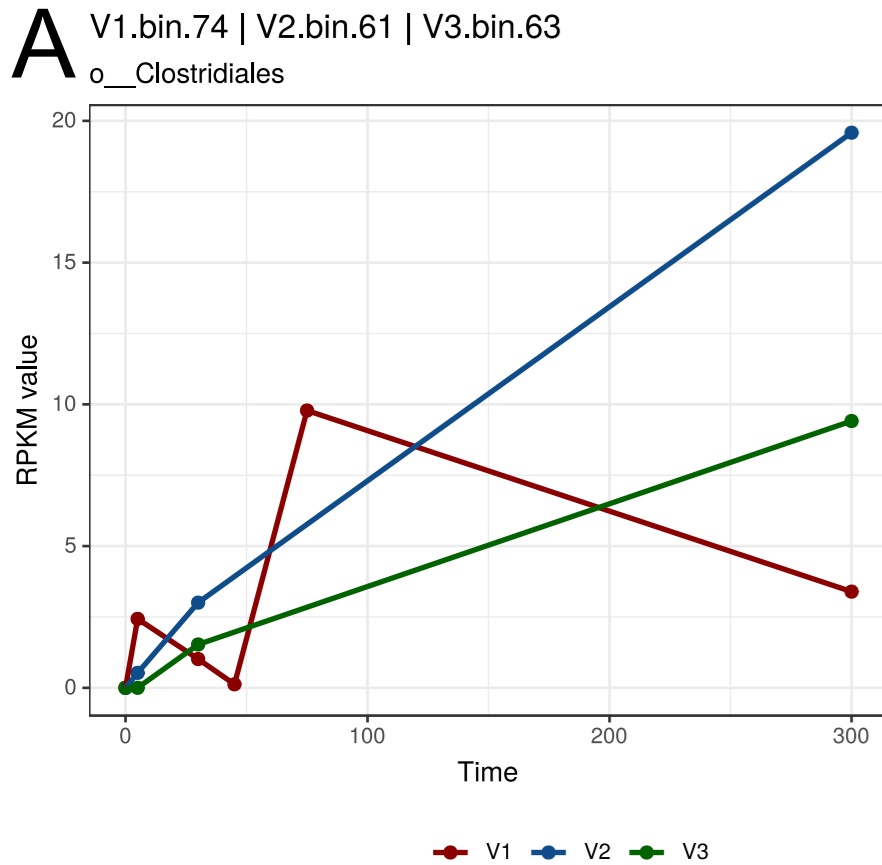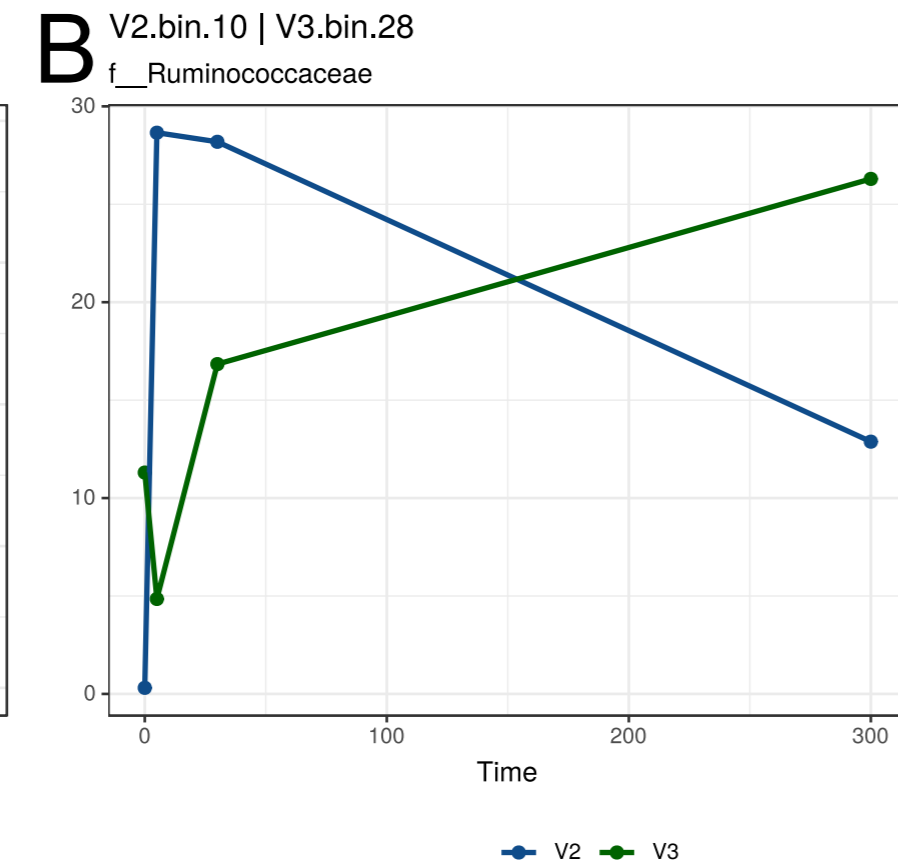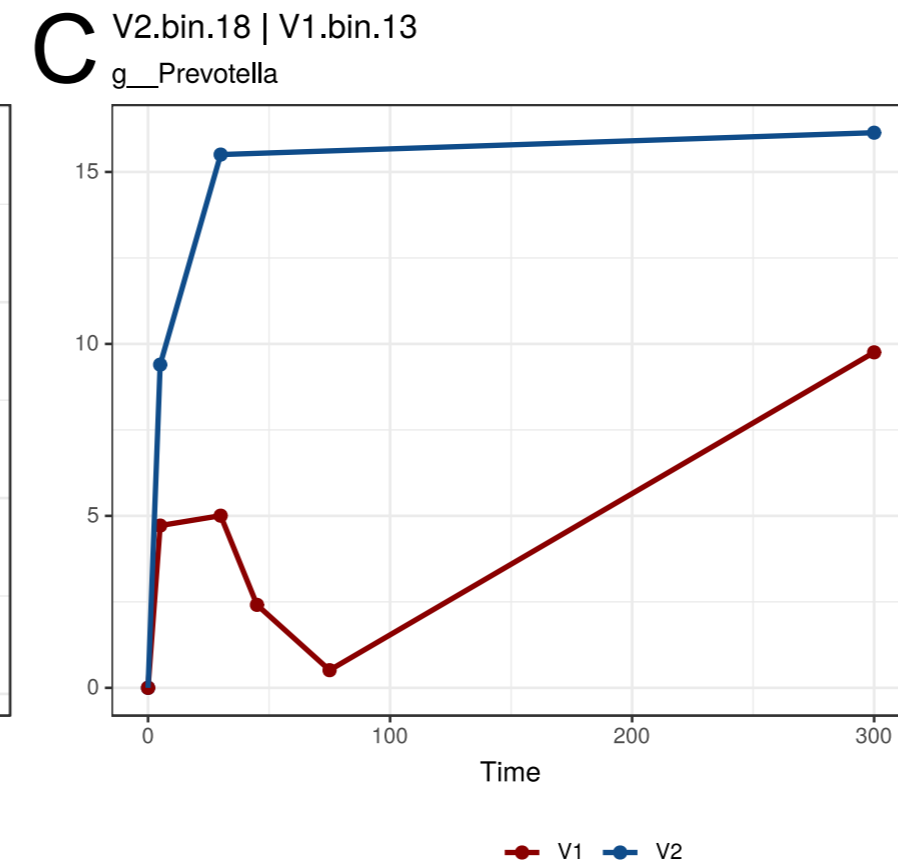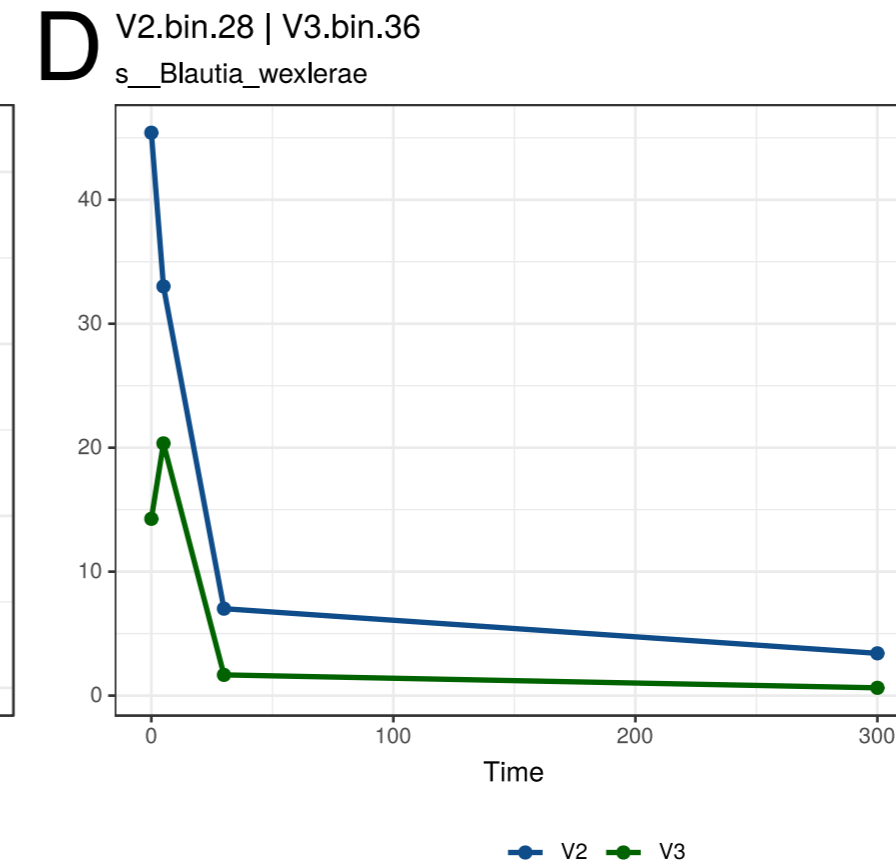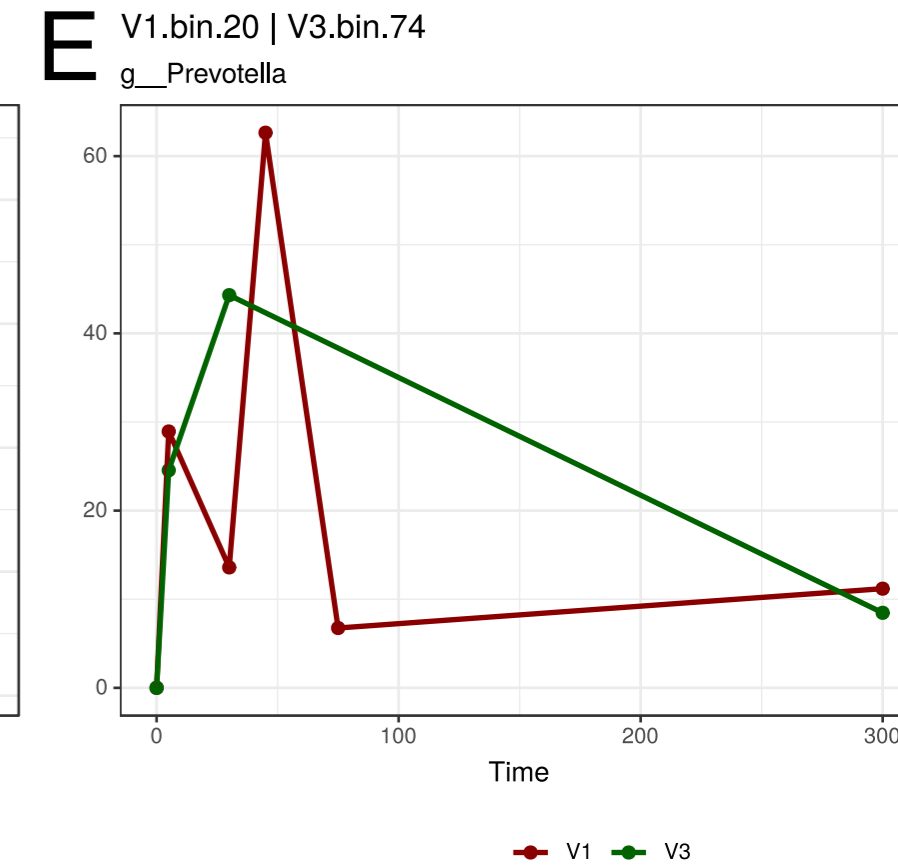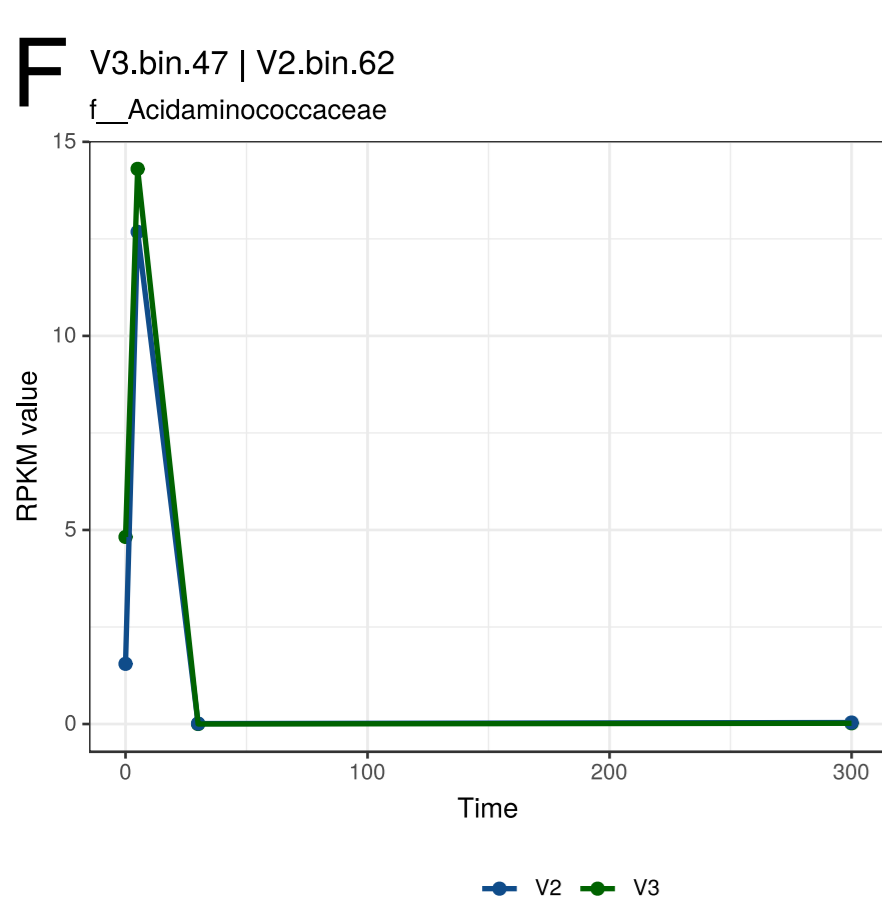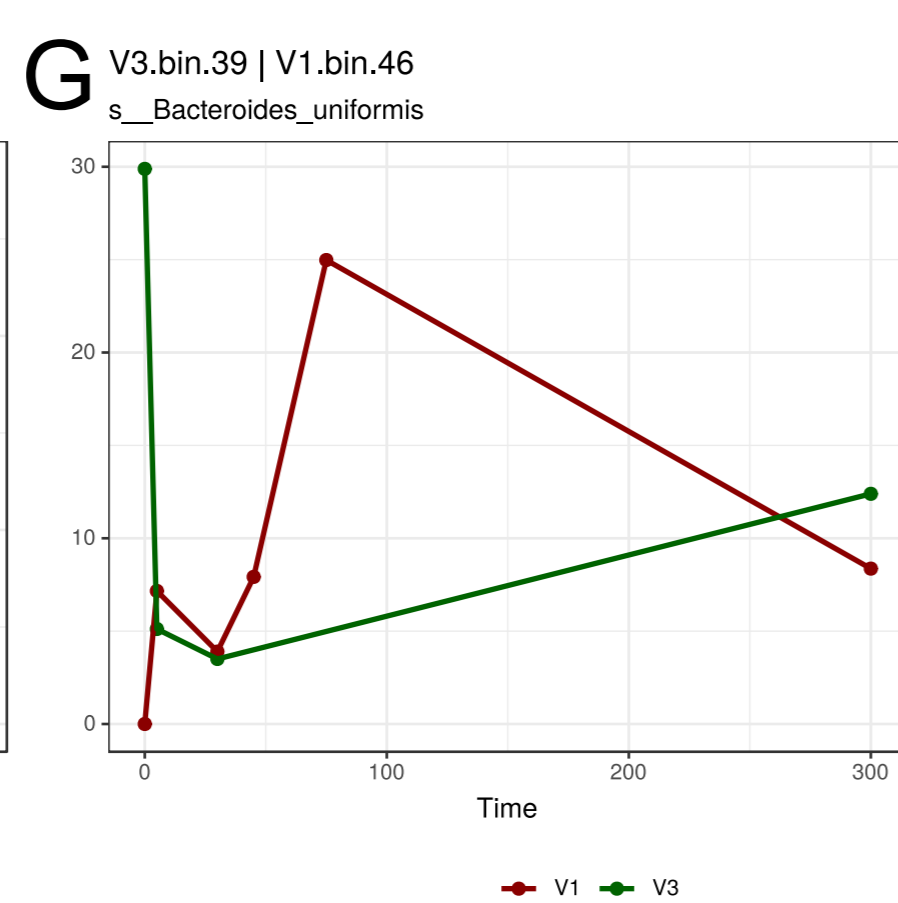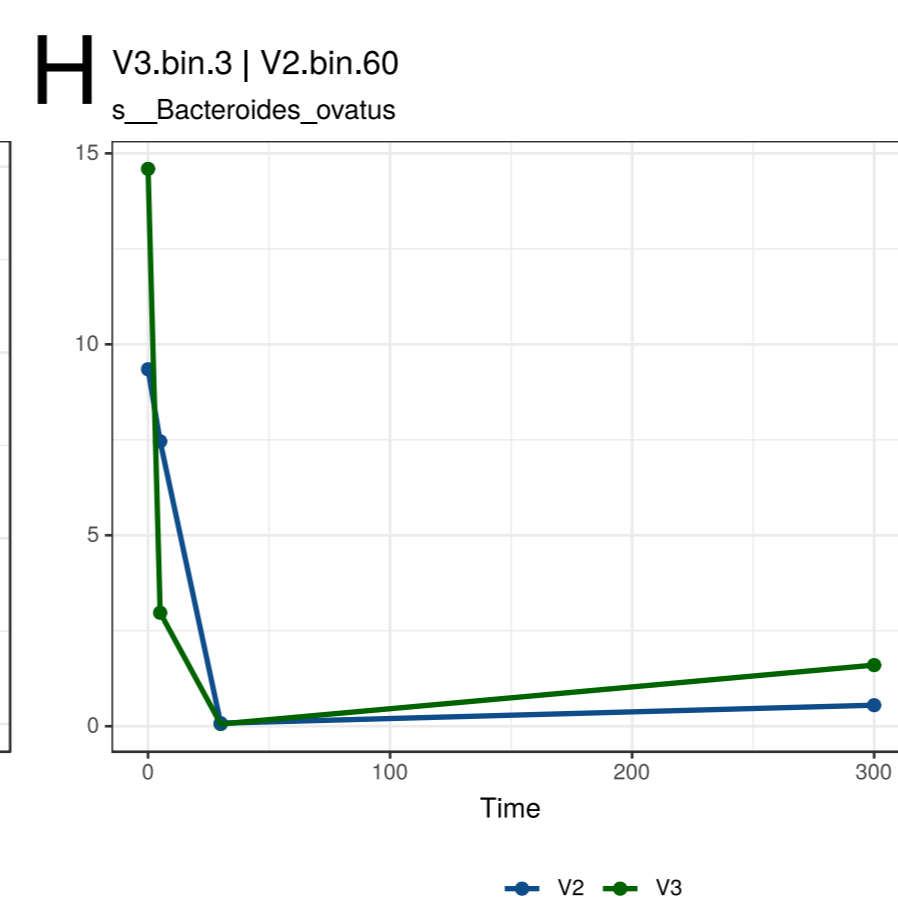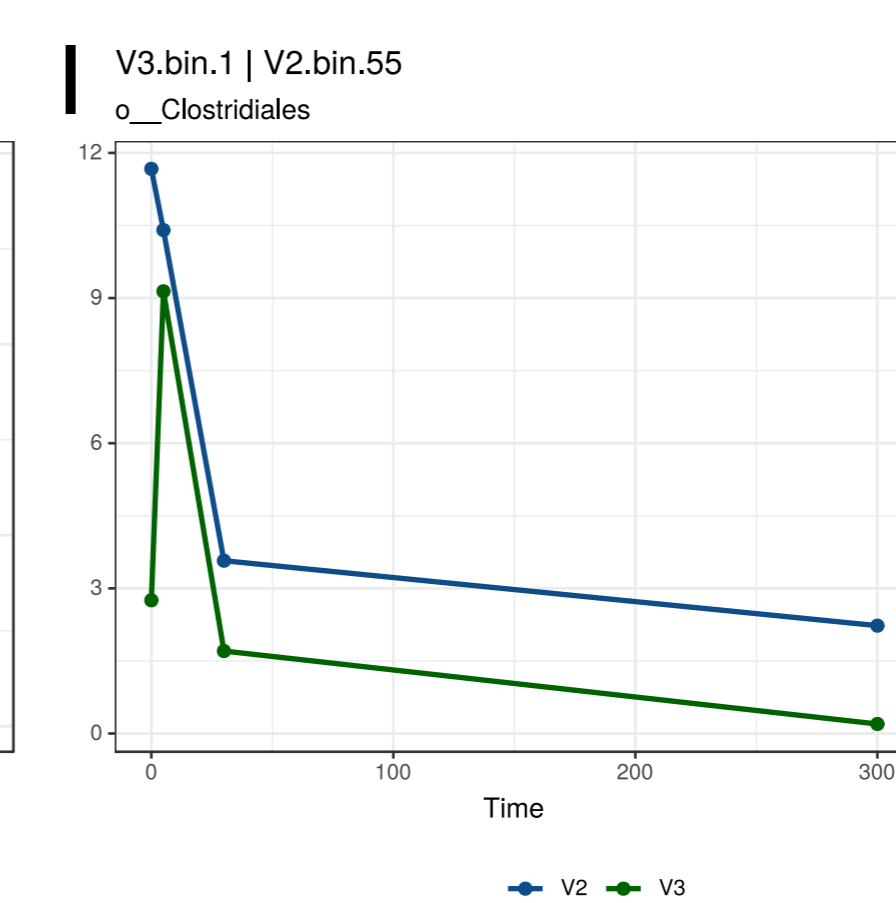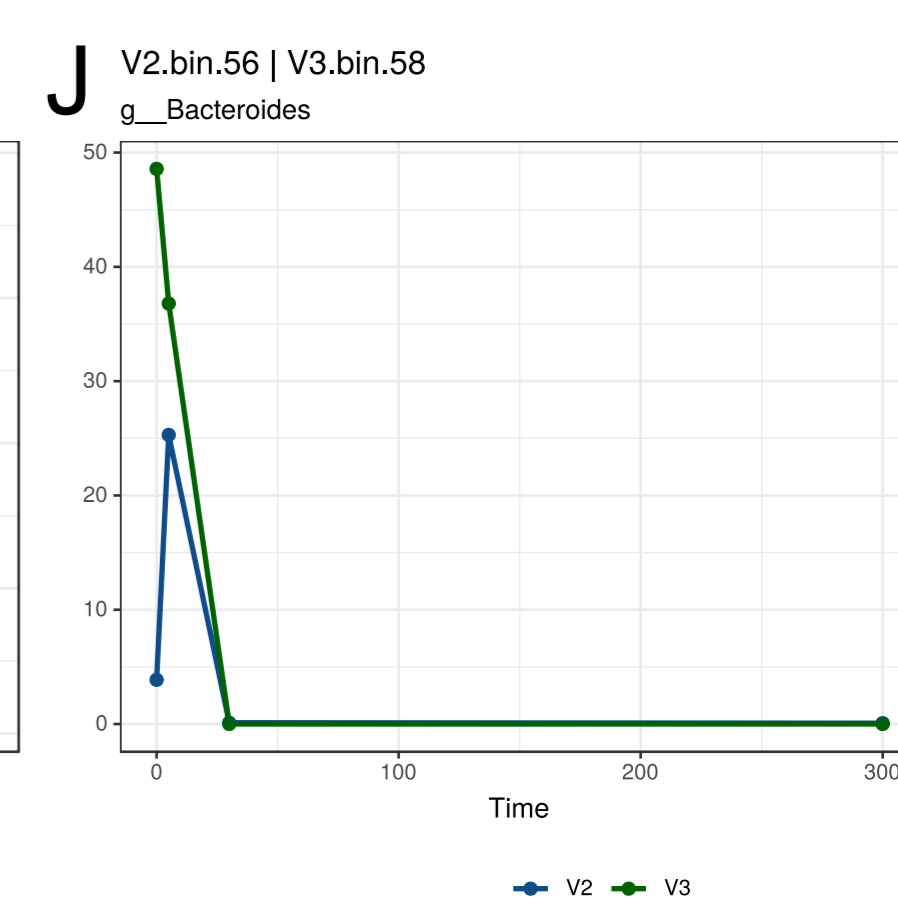

Supplement: Supplementary file 3 — Additional file 3 Additional file 3: Figure S3 Similar recipient MAGs (with 100% similarity of 43 marker proteins) relative abundance change. [file 12866_2019_1689_MOESM3_ESM.pdf]

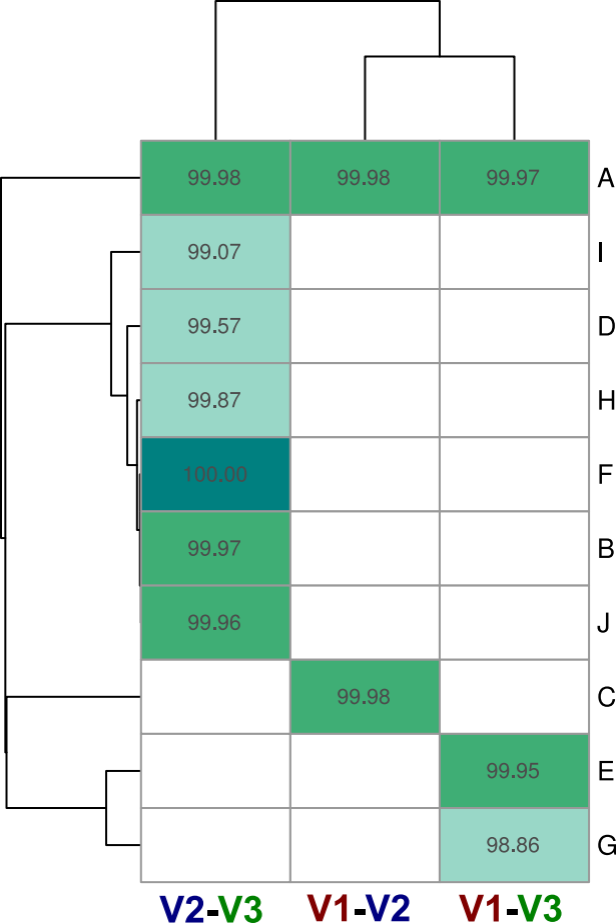

Supplement: Supplementary file 4 — Additional file 4 Additional file 4: Figure S4 The ANI between similar recipient MAGs. Recipient MAGs with 100% AA similarity of 43 marker proteins were selected. [file 12866_2019_1689_MOESM4_ESM.pdf]

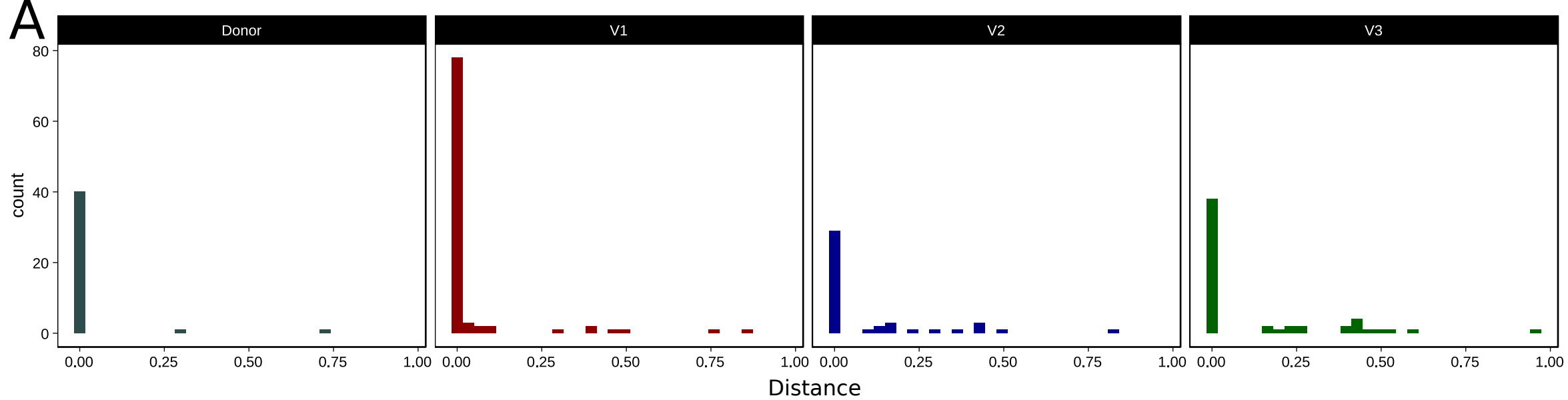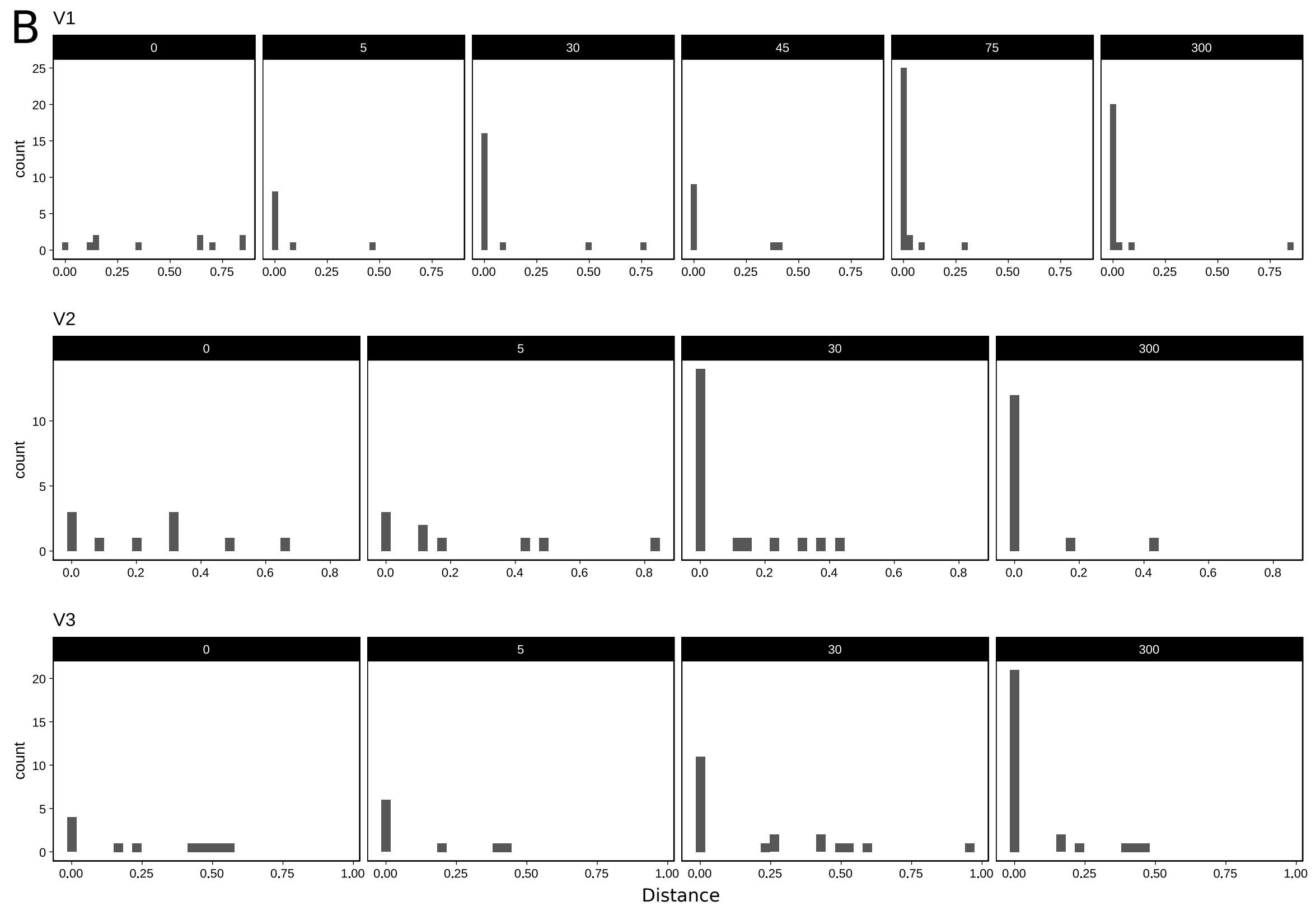

Supplement: Supplementary file 5 — Additional file 5 Additional file 5: Figure S5 Comparison of similarity donor and recipient bacteria based on metaSNV profiling. (A) Histograms shows frequency of distances between major donor sample (used for FMT procedure), additional donor samples and recipient metagenomes. Metagenomic samples obtained before FMT were removed. (B) Evolution of distances between major donor sample and metagenomic samples of each recipient over time. [file 12866_2019_1689_MOESM5_ESM.pdf]
